# Dynamics of gut resistome and mobilome in early life: a meta-analysis

**DOI:** 10.1016/j.ebiom.2025.105630

**Published:** 2025-03-05

**Authors:** Ahmed Bargheet, Hanna Theodora Noordzij, Alise J. Ponsero, Ching Jian, Katri Korpela, Mireia Valles-Colomer, Justine Debelius, Alexander Kurilshikov, Veronika Kuchařová Pettersen

**Affiliations:** a Host-Microbe Interaction Research Group, Department of Medical Biology, UiT The Arctic University of Norway, Tromsø, Norway; b Center for New Antibacterial Strategies, UiT The Arctic University of Norway, Tromsø, Norway; c Centre for Ecological and Evolutionary Synthesis, Department of Biosciences, University of Oslo, Norway; d Human Microbiome Research Program, Faculty of Medicine, University of Helsinki, Finland; e Department of Medical and Life Sciences, Pompeu Fabra University, Barcelona, Spain; f Department CIBIO, University of Trento, Italy; g Department of Epidemiology, Johns Hopkins Bloomberg School of Public Health, Baltimore, MD, USA; h Department of Genetics, University of Groningen, University Medical Center Groningen, the Netherlands

**Keywords:** Bacterial drug resistance, Mobile genetic elements, Plasmids, Gut microbiota, *Escherichia coli*, Infant, Cohort studies, Metagenomics, Meta-analysis

## Abstract

**Background:**

The gut microbiota of infants harbours a higher proportion of antibiotic resistance genes (ARGs) compared to adults, even in infants never exposed to antibiotics. Our study aims to elucidate this phenomenon by analysing how different perinatal factors influence the presence of ARGs, mobile genetic elements (MGEs), and their bacterial hosts in the infant gut.

**Methods:**

We searched MEDLINE and Embase up to April 3rd, 2023, for studies reporting infant cohorts with shotgun metagenomic sequencing of stool samples. The systematic search identified 14 longitudinal infant cohorts from 10 countries across three continents, featuring publicly available sequencing data with corresponding metadata. For subsequent integrative bioinformatic analyses, we used 3981 high-quality metagenomic samples from 1270 infants and 415 mothers.

**Findings:**

We identified distinct trajectories of the resistome and mobilome associated with birth mode, gestational age, antibiotic use, and geographical location. Geographical variation was exemplified by differences between cohorts from Europe, Southern Africa, and Northern America, which showed variation in both diversity and abundance of ARGs. On the other hand, we did not detect a significant impact of breastfeeding on the infants' gut resistome. More than half of detected ARGs co-localised with plasmids in key bacterial hosts, such as *Escherichia coli* and *Enterococcus faecalis*. These ARG-associated plasmids were gradually lost during infancy. We also demonstrate that *E. coli* role as a primary modulator of the infant gut resistome and mobilome is facilitated by its increased abundance and strain diversity compared to adults.

**Interpretation:**

Birth mode, gestational age, antibiotic exposure, and geographical location significantly influence the development of the infant gut resistome and mobilome. A reduction *in E. coli* relative abundance over time appears as a key factor driving the decrease in both resistome and plasmid relative abundance as infants grow.

**Funding:**

Centre for Advanced Study in Oslo, Norway. Centre for New Antibacterial Strategies through the 10.13039/100016934Tromsø Research Foundation, Norway.

**Research in context**

**Evidence before this study**

Infants, with their developing immune systems and common exposure to hospital environment during birth, are particularly susceptible to antibiotic-resistant infections. Our previous research showed that low gestational age, combined with frequent antibiotic use, negatively alters early life resistome and mobilome, leading to an increased gut carriage of antibiotic resistant genes (ARGs) and mobile genetic elements (MGEs). Another small-scale study suggested that infants delivered by Caesarean section have lower ARG diversity but at a higher abundance when compared to vaginally delivered infants. In addition, several studies identified *Escherichia coli* as a prominent bacterial host of ARGs in the infant gut. However, none of these associations have been confirmed in larger cohorts, including careful considerations of potential confounding factors.

**Added value of this study**

Previous studies provided valuable but fragmented insights in terms of how perinatal factors influence the infant gut resistome. We employed a meta-analytical approach using metagenomic data derived from 3497 stool samples of 1270 infants. In this way, the meta-analysis reduced biases inherent to individual studies due to low sample sizes and allowed a statistically robust description of the effects of antibiotic use, gestational age, delivery mode, feeding type, and geography on the infants’ gut resistome and mobilome. This study reports longitudinal trajectories of both resistome and mobilome abundance and diversity, as well as provides insights into how strain level dynamics of the *E. coli* species modulate the infant resistome.

**Implications of all the available evidence**

To mitigate the development and spread of antibiotic resistance, factors promoting infant gut resistome, and in particular the impact of antibiotic use, should be considered in clinical practices around childbirth and neonatal care. Geographical differences in the gut resistome diversity and abundance highlight the need for region-specific public health strategies. Further research should focus on determining ARG transmission pathways and how resistant pathogenic strains emerge.

## Introduction

In the realm of human microbial ecology, studying the gut carriage of antibiotic resistant genes (ARGs) in relation to potential sources of resistant organisms/resistance genes and factors that modify their carriage addresses one of the top global public health and development threats: antimicrobial resistance (AMR). AMR currently stands as a leading cause of mortality worldwide, particularly in low-resource regions.^1^^,^^2^ Understanding its reservoirs, including the human gut microbiota, is a key component of strategies to prevent the spread of AMR.

Intriguingly, the infant gut microbiota exhibits a relatively higher carriage of ARGs than that of adults, even for infants never exposed to antibiotics.^3^ Among the hypothesised explanations is the higher microbiota diversity and, consequently, increased colonisation resistance in adults compared to infants, in addition to a lower relative abundance of *Escherichia coli*, a typical bacterial host of ARGs in the human gut.^4^ The relative abundance of *E. coli* likely explains much of the associations that have been reported between infant gut resistome and different exposures.^5^ Nevertheless, besides a consistent observation linking elevated ARG levels with lower gestational age and antibiotic use,^6^^,^^7^ evidence is limited on the influence of birth mode,^8^ different feeding types,^7^ and geography on the gut resistome.

The gut mobilome, comprised of mobile genetic elements (MGEs) such as plasmids, transposons, and integrons, plays a crucial role in spreading ARGs between opportunistic pathogens, true pathogens, commensals, and other symbiotic bacteria. A key question is to what extent antibiotic use and other factors affect the abundance and diversity of MGEs within the gut microbiota. These scattered insights led us to comprehensively investigate the dynamics of the infant gut resistome and mobilome in relation to factors commonly encountered during infancy. Our methodology included a systematic selection of previously published data sets and a combination of primary data through a subject-level meta-analysis framework.^9^ We focused on the influence of birth mode, feeding type, gestational age, antibiotic use, and geography, in addition to highlighting the attributes of *E. coli* species that are behind its prominent role in the infant gut resistome.

## Methods

### Ethics

Ethical approval and consent to participate were not applicable as this was a meta-analysis of already conducted studies. All included studies had reported corresponding approvals from ethical committees, and had publicly accessible the sequencing data and metadata, allowing for data re-analysis. We did not include any studies which required additional ethical approvals or authorisations for access of the data.

### Systematic search strategy and data extraction

We conducted literature searches in two high-quality databases (MEDLINE and Embase) up to April 3rd, 2023, using several MeSH terms and appropriate free-text search words (Figure S1). After collating the identified studies, we removed review articles and duplicates/triplicates and ended up with 677 studies to screen.

The screening of the studies was performed using Covidence systematic review software (Veritas Health Innovation, Melbourne, Australia, www.covidence.org). The titles and abstracts were independently considered for eligibility by two researchers according to predetermined inclusion and exclusion criteria, while a third author had the decisive vote in case of a conflict. Inclusion criteria were original reports on gut/faecal microbiota of human infants and potentially their mothers that used shotgun metagenomics, written in English and published in the last ten years. The 10-year look-back window captured a vast majority of relevant studies since shotgun metagenomic sequencing became more widely accessible to researchers after the introduction of Illumina sequencing systems at the beginning of the 2010s. In addition to the fact that very few metagenomic studies on infant microbiome existed before 2013, using studies from the past decade allowed better standardisation of the data, minimising potential biases from studies with older methodologies that differed significantly.^10^ We excluded reviews, editorial letters, conference abstracts, books, book chapters and commentaries, animal and environmental studies, meta-analyses that used only published datasets, and studies on skin, lung, vaginal, breastmilk, and microbiota other than from the gut/faeces. Studies using 16S rRNA gene sequence analysis or enrichment techniques were also excluded, similar to studies that primarily included disease cohorts (*i.e*., cohorts investigating the impact of infant genetic and symptomatic diseases on the gut microbiota). Finally, we excluded studies where we could not access the raw sequencing data and associated metadata. The systematic search has been registered in the International Prospective Register of Systematic Reviews, PROSPERO, with the ID CRD42024607114.

Following the search, corresponding metagenomic sequences were collected from data depositories (SRA-NCBI, ENA). These metagenomes were retrieved from 14 cohorts^6^^,^^8^^,^^11^, ^12^, ^13^, ^14^, ^15^, ^16^, ^17^, ^18^, ^19^, ^20^, ^21^, ^22^ representing samples from 10 countries (USA, UK, Norway, Netherlands, Sweden, Finland, Luxemburg, Italy, South Africa, Zimbabwe) and using Illumina sequencing platforms (Table S1).

### Metadata curation

The associated metadata were manually curated (Table S2). For infant samples, the following minimum metadata were required: age at sampling, birth mode, feeding type (breastfeeding, formula feeding, and solid food), prematurity status (gestational age less than 37 weeks), and antibiotic exposures. Studies that were missing the required metadata were excluded altogether. When solid food status was unknown, it was assumed that infants did not receive solid food before three months, and solid foods were introduced by nine months of age. However, we did not make any assumptions regarding breast- or bottle-feeding, *i.e*., we used the information on milk feeding exactly as reported in individual studies. Infant metadata concerning antibiotic exposures were curated to generate two Boolean variables (Table S3): (1) capturing if the infant had one or more antibiotic exposures before the sample collection, and (2) describing if the infant was receiving an antibiotic treatment at the time of sample collection. A single antibiotic exposure reported before the sampling point was considered as antibiotic exposure, excluding possible exposures through intrapartum antibiotic treatments, as this information was missing in all cohorts except Matharu et al., 2022. For one cohort including samples from twin pairs, only one of the twin infants was selected according to the number of samples available. After metadata curation, cohorts with less than 20 infant samples available were excluded.

### Bioinformatics pre-processing

Metagenomic reads were quality-controlled using FastQC (v.0.11.9).^23^ Sequences of poor quality and adaptor regions were filtered out using Trimmomatic (v.0.39).^24^ The parameters employed were: *java -jar trimmomatic-0.39.jar PE -phred33 ILLUMINACLIP:NexteraPE-PE.fa:2:30:10 LEADING:3 TRAILING:3 SLIDINGWINDOW:4:15 MINLEN:36*. To eliminate human DNA contaminants, sequences mapping to the human reference genome (GRCh38, downloaded from NCBI GenBank) were filtered out utilising Bowtie2 (v.2.4.4),^25^ employing the *--very-sensitive -- end-to-end* parameters. The non-human-aligned reads were isolated via SAMtools, (v.1.12),^26^ utilising flags *-f 12 -F 256*, and these reads were earmarked for inclusion in all downstream analyses. Metagenomes with less than 1 million reads after quality control were excluded from the analysis. The read statistics for the samples included in this analysis are detailed in Table S1.

### Reference-based taxonomic, resistome, and mobilome profiling

We employed the following reference-based approaches, relying on reference databases. Taxonomic profiling was obtained using MetaPhlAn4 v.4.0.6^27^ against the CHOCOphLAN database. Metagenomes with more than 50% unclassified reads were excluded from the analysis to ensure taxonomic reliability (Figure S2). The relative abundance of ARGs was quantified by employing ShortBRED v.0.9.5.^28^ Resistance-related determinants sourced from the CARD database, v.3.2.7^29^ served as the proteins of interest for identifying marker families using ‘*shortbred_identify.py’* with *‘--clustid 0.95’* option. ARG abundances were normalised in reads per kilobase reference per million mapped reads (RPKM) using ‘*shortbred_quantify.py’*. Specifically, ARGs detected in a sample were normalised to the total number of reads in a sample and the gene length. Similarly, the normalised relative abundances of MGEs, such as transposases, integrases, recombinases, and integrons, were ascertained using ShortBRED against a curated reference database of MGEs, MobileOG v.1.6.^30^ The clinical relevance of individual ARGs was based on the AMR Gene Family previously reported as clinically relevant by Diebold et al.^31^ and the National Database of Antibiotic Resistant Organisms (https://www.ncbi.nlm.nih.gov/pathogens/antimicrobial-resistance/). We excluded ARGs encoding efflux pumps and metabolic functions due to the difficulties of distinguishing their physiologic role from their role in AMR, and the necessity for accurate identifications of specific mutant variants conferring AMR. Moreover, when assessing the carriage of ARGs by specific bacterial hosts, we checked if the identified ARGs have clinical relevance in the linked bacterial species. Strain analysis was performed using the StrainGST from StrainGE v.1.3.3^32^ upon downloading the NCBI reference genomes for *E. coli*.

### Assembly-based taxonomic, resistome, and mobilome profiling

The assembly-based approach consisted of assembling the quality-filtered short-read sequences into longer contiguous sequences (contigs) using MEGAHIT v.1.2.9^33^ with the default parameters. For the assessment of assemblies, MetaQUAST from QUAST v.5.2.0^34^ was used with the *-m* 1000 option (Table S4). The contigs were then annotated against the CARD database using ABRicate v.1.0.1,^35^ applying parameters *–minid 80 and –mincov 80*. Subsequently, Open Reading Frames (ORFs) were predicted from contigs exceeding 1000 base pairs, which harboured ARGs identified by ABRicate against CARD, employing Prodigal v.2.6.3^36^ with the default parameters. Following this, we determined the taxonomy by comparing amino acid sequences from Prodigal against the NCBI non-redundant database, executed using DIAMOND v. 2.1.6^37^ with the following options: *‘--evalue 0.00001’*, *‘--id 95’,* and *‘--query-cover 95’*. For contigs assigned to multiple species, we retained only those where at least 75% of ORFs were assigned to a single species. Similarly, MGEs were identified by aligning amino acid sequences from Prodigal against the MobileOG database using DIAMOND. The co-localisation analysis was limited to contigs explicitly associated with ARGs, and those linked to ARGs without assignment to specific bacteria were excluded. To facilitate accurate comparisons, we normalised the counts of ARGs and MGEs to sequencing depth. We did this by dividing each count by the sample's total read count and multiplying the result by one million.

### Statistics

#### Linear mixed models for α-diversity and differential abundance analyses

α-diversity of taxonomy, resistome and mobilome was analysed by using *vegan* v.2.5.7 R package.^38^ We estimated the trajectory of α-diversity for resistome and mobilome using a linear mixed model employing the *lmerTest* v.3.1.3^39^ R package, with ‘infant age’, ‘bead-beating’, and various ‘sequencing platforms’ as fixed effects. We included ‘subject_id’ nested within ‘study_name’ as a random intercept effect. Only complete cases, *i.e*., samples with the required metadata, were used (*n* = 3497; 87.8%). The *lmerTest* package was also used to evaluate the statistical influence of variables on the diversity and relative abundance of the resistome and mobilome. This way, the models accounted for the batch effects (absence/presence of bead beating step and differences in Illumina sequencing platforms) and multiple samples from the same individual and between-study heterogeneity (‘subject id’ nested within ‘study name’). The antibiotic variable used in all models reflects whether an infant received at least one antibiotic treatment before the sampling event. To standardise resistome and mobilome relative abundance, we applied an inverse-normal transformation (*qnorm* function in R). Additionally, we examined collinearity among variables using the variance inflation factor, and a threshold of 8 was pre-specified as indicative of problematic collinearity. To identify differentially abundant features, we utilised the *ANCOM-BC* v.2.2.2^40^ R package with the Benjamini-Hochberg procedure for *p*-values adjustment. In this model, we also considered study-dependent batch effects and ‘subject ID’ and ‘study name’ as random effects.

#### β-diversity, ordination, and community typing analyses

The analysis of β-diversity was executed using the *“vegdist”* function within the *vegan* v.2.5.7^38^ R package, applying principal coordinate analysis (PCoA) along with Bray–Curtis dissimilarity indexes. To ascertain the statistical significance of composition differences, we conducted permutational multivariate analysis of variance (PERMANOVA) using the *'adonis'* function of the *vegan* package, with 999 permutations. Procrustes analysis was employed to explore correlations between taxonomic composition, resistome, and mobilome. For this, PCoA ordinations of ARGs, MGEs, and taxonomic compositions were scaled and rotated uniformly to minimise their squared differences. We computed the symmetric Procrustes correlation coefficients and statistics using the ‘*procrustes*’ and *‘protest’* functions from the *vegan* v.2.5.7^38^ R package, applying 9999 permutations. Community typing analysis was carried out employing non-negative matrix factorisation (NMF), facilitated by the *NMF* v.0.26^41^ R package, as previously described.^42^

#### Other statistical analyses and data visualisation

The statistical analysis was conducted using R software v.4.3.1. Graphical illustrations were mainly created with the *ggplot2* v.3.3.6 package.^43^ The heatmaps were visualised using *pheatmap* v.1.0.12 package.^44^ We identified variable importance using the *vip* (v.0.4.1) R package^45^ after running a Random Forest model with the *randomForest* (v.4.7.1.2) R package.^46^ The *I*^*2*^ statistic was applied to quantify the proportion of variability in bacterial diversity (Shannon index) between studies, using the *rma* function from the *metafor* (v.4.6.0) R package.^47^ Statistical significance was defined as *p* < 0.05.

### Role of funders

The funders had no role in study design, data collection, data analyses, interpretation, or writing of the report.

## Results

### A dataset overview and factors influencing taxonomical variation

**Fig. 1:**
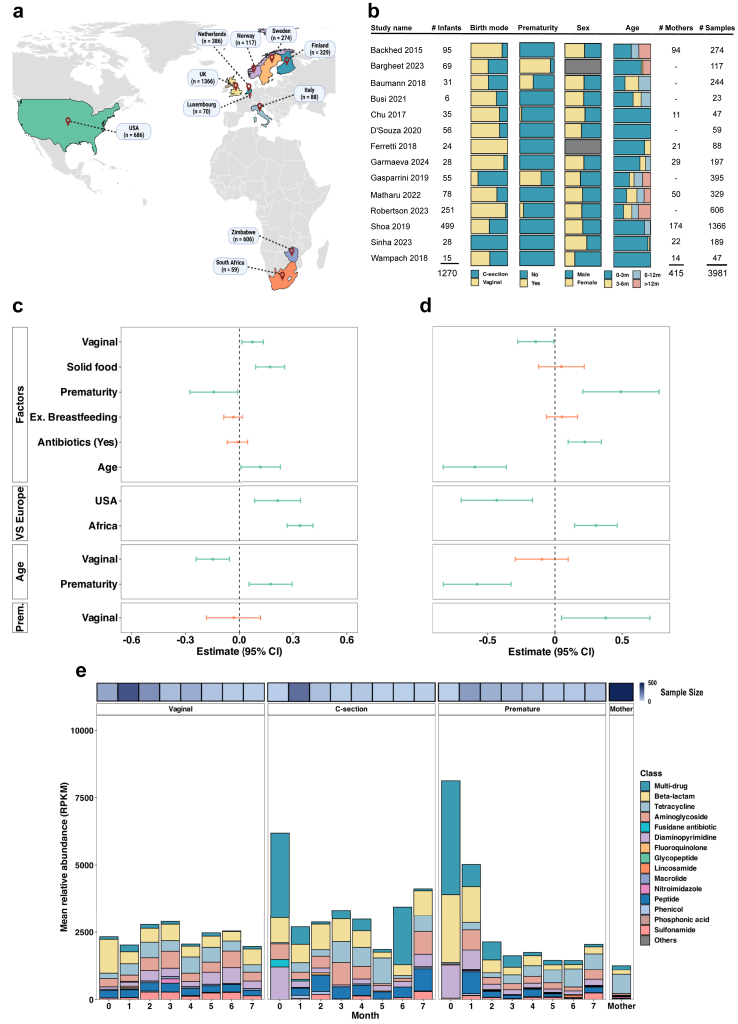
**Infant gut resistome dynamics.** (a) Map indicating the geographical locations of included cohorts. (b) Overview of the cohorts' characteristics. A grey bar in panel B denotes missing data. The impact of selected variables on the infant gut resistome α-diversity (c) and abundance (d), as determined by a linear mixed effect modelling (LMM) (*n* = 3497 samples from 1270 infants). The fixed effects used in LMM included infant age, the presence/absence of bead-beating, and the use of various sequencing platforms. The estimates are in Confidence Interval (CI) of 95% as determined by LMM. The green error bars indicate statistically significant results (*p* < 0.05), while the orange error bars represent non-significant results (*p* > 0.05). (e) The relative abundance of the gut resistome quantified in reads per kilobase per million mapped reads (RPKM). The main antibiotic resistant genes families in three selected infant groups as compared to maternal samples. The ‘C-section’ (*n* = 330 samples from 173 infants) and ‘Vaginal’ groups (*n* = 750 samples from 513 infants) are full-term infants that were exclusively breastfed the first three months of life and not exposed to antibiotics during the displayed period. The ‘Premature’ group (*n* = 372 samples from 135 infants) is preterm babies without discrimination of birth mode, feeding, or exposure to antibiotics. In addition, 484 maternal samples were used in the analysis. Multi-drug is ARG conferring resistance to two or more antibiotic classes.

A systematic literature search of infant cohorts, which included shotgun metagenomic sequencing of stool samples, identified 14 studies^6^^,^^8^^,^^11^, ^12^, ^13^, ^14^, ^15^, ^16^, ^17^, ^18^, ^19^, ^20^, ^21^, ^22^ (Fig. 1a). We focused on 3981 high-quality metagenomic samples (over 1 million sequencing reads each) from infants (3497 samples from 1270 individuals) and their mothers (484 samples from 415 individuals). The dataset includes a majority of infants delivered vaginally (≈64%, *n* = 816) and born at term (≈87%, *n* = 1101), with balanced sex distribution (≈45% female, *n* = 566) (Fig. 1b). While including infant samples collected between birth and two years of age, 1843 infant samples (≈59%, *n* = 1064) were collected between birth and three months of age (Figure S3a). The infant feeding information was curated to cover breastfeeding, formula feeding, and/or solid food at the time of sampling (Figure S3b). Additionally, exposure to antibiotics prior to the sampling time was curated for all infant samples (Figure S3c).

Because the meta-analysis included multiple cohorts, it was necessary first to identify potential confounding variables and individual cohort biases before downstream analyses. We initially used the *I*^*2*^ statistic to estimate the proportion of variability in microbiota α-diversity (Shannon index) across studies attributable to within-sample microbial diversity. The analysis revealed substantial heterogeneity among cohorts (*Tau*^*2*^ = 0.15, standard error SE = 0.059; *I*^*2*^ = 97.81%, *p* = 0.0001; Figure S4a). This variance in α-diversity was mainly driven by differences in the infant age across the studies (Figure S4b).

We next examine the differences in microbial community composition using β-diversity analysis, which accounted for multivariate compositional patterns and confounding factors. The study effect explained 4% of the species-level taxonomic variance (R^2^) in infant samples for models including sequencing depth and sampling age (*p* = 0.001, adonis2 PERMANOVA marginal effects of terms analysis on Bray–Curtis; permutations = 999, Table S5). Other technical factors that were significantly associated with the taxonomic β-diversity of the samples included the use of a bead-beating step in the DNA extraction (R^2^ = 1%, *p* = 0.001), the region of sample collection (Europe, North America, or Southern Africa, R^2^ = 2%, *p* = 0.001) and the choice of sequencing platform (R^2^ = 1%, *p* = 0.001). However, the impact of these variables was reduced for models accounting for study identity, with geographical region and extraction method not significant and sequencing platform accounting for less than 0.005% of the variance (*p* = 0.012). Further, the β-diversity of infant samples was significantly associated with birth mode (R^2^ = 1%, *p* = 0.001), feeding practices (R^2^ = 2%, *p* = 0.001), and prior infant exposures to antibiotics (R^2^ = 0.2%, *p* = 0.001), in models accounting for sequencing depth, infant age, and study identity. Additionally, we ran a Random Forest model to identify the contribution of each predictor to the bacterial diversity, which corroborated the PERMANOVA results (Figure S4c). We then used the information on which factors significantly influence taxonomic variation in subsequent linear mixed effect (LMM) modelling to account for the inter-study heterogeneity due to batch effects (absence/presence of bead beating step and differences in Illumina sequencing platforms) and to control for multiple samples from the same individual.

### The impact of early-life factors on the development of infant gut resistome

Using a reference-based pipeline for resistome profiling, we detected 362 ARGs (Table S6) and refined this list to 197 clinically relevant ARGs. In the subsequent LMM analysis of the infant gut resistome dynamics, we first run a sensitivity analysis of a subset of all studies. Specifically, we considered that the African studies include children exposed to HIV in utero and with potentially different exposure to medications than in the other cohorts. The sensitivity analysis of running the same statistical model with and without the African cohorts identify minor differences in significant levels of the selected variables (Table S7). Specifically, exclusion of the African cohorts affected the solid food variable, becoming non-significant in its effect on the resistome α-diversity. On the other hand, the effect of being born vaginally on resistome abundance decreased significantly with age upon exclusion of the African cohorts. Significance levels of other variables have not changed, confirming the model robustness in accounting for variability between studies and adjusting for confounding by the main factors.

Results of the LMM analysis that included all cohorts revealed that vaginally delivered infants have higher ARG α-diversity but lower total relative abundance compared to C-section infants (Fig. 1c). ARG diversity was significantly modulated by gestational age and the introduction of solid food (LMM; *p* < 0.01). Unsurprisingly, antibiotic exposure was strongly linked to increased ARG relative abundance (LMM; *p* < 0.01). Geographical differences showed higher ARG diversity in infants from the USA and African cohorts (Zimbabwe and South Africa) compared to European infants (LMM; *p* < 0.01, Fig. 1c). However, while infants from African cohorts also had a higher ARG relative abundance than infants from European cohorts, the US cohorts displayed an opposite trend, that is, a significantly lower ARG abundance (LMM; *p* < 0.01, Fig. 1d). Lastly, resistome composition exhibited greater variability among younger neonates, which converged to more similar over time (adonis2, Bray–Curtis; *p* < 0.001; premutation 999; Figure S5a).

To characterise the different resistome trajectories, we defined three infant groups and followed them over the first seven months, given by the sample/data availability within the 3981 meta-analysis samples (Figure S3d). The groups included term-born infants delivered by C-section (‘C-section’ group: 330 samples from 173 infants) or vaginally (‘Vaginal’ group: 750 samples from 513 infants), both exclusively breastfed for the first three months, without antibiotic exposure after birth, but with unknown maternal perinatal exposures. This grouping allowed us to delineate the effect of birth mode. To assess the impact of gestational age, we included prematurely born infants (‘Premature’ group: 372 samples from 135 infants) without discriminating birth mode, feeding, or antibiotic exposure. For the latter group, we considered that the effect of birth mode is minimal in premature infants,^48^ the feeding of these infants was mixed (including both formula and breastmilk), and most preterm infants had at least one reported antibiotic exposure during the first three months of life (71.8%).

The LMM analysis highlighted age as a significant driver of the gut resistome profile. Immediately after birth, β-lactamases were the most abundant ARG class in the ‘Vaginal’ group, followed by genes conferring resistance to aminoglycosides and peptides (Fig. 1e). Although non-significant, the ‘C-section’ and ‘Premature’ groups tended to have higher levels of multi-drug resistance ARGs early on. Over time, preterm infants showed a notable decrease in β-lactamase abundance and an increase in tetracycline resistance genes, aligning with levels seen in mothers. Additionally, C-section infants had higher levels of diaminopyrimidine ARGs and lower levels of β-lactam ARGs in the first three months of life (Benjamini-Hochberg adjusted *p* < 0.01; Figure S6a). All infant groups had a higher ARG relative abundance at birth compared to adults (Kruskal–Wallis followed by Dunn's test; mean adj. *p* = 0.003), and the ‘Vaginal’ group did not show a significant decrease in ARGs during the first seven months.

Stratifying infants according to the gestational age, diet, birth mode, and antibiotic exposure underlined that the gut resistome profile is a combined outcome of multiple factors that determine its trajectory (Figure S6b). When examining the resistome trends in all full-term infants (2985 samples of 1131 infants, including different diets, birth modes, and medication exposure) up to 14 months of age, the ARG diversity increased, and relative abundance decreased (Figure S6c and d, respectively; LMM; *p* < 0.01).

### Developmental dynamics of infant gut mobilome

**Fig. 2:**
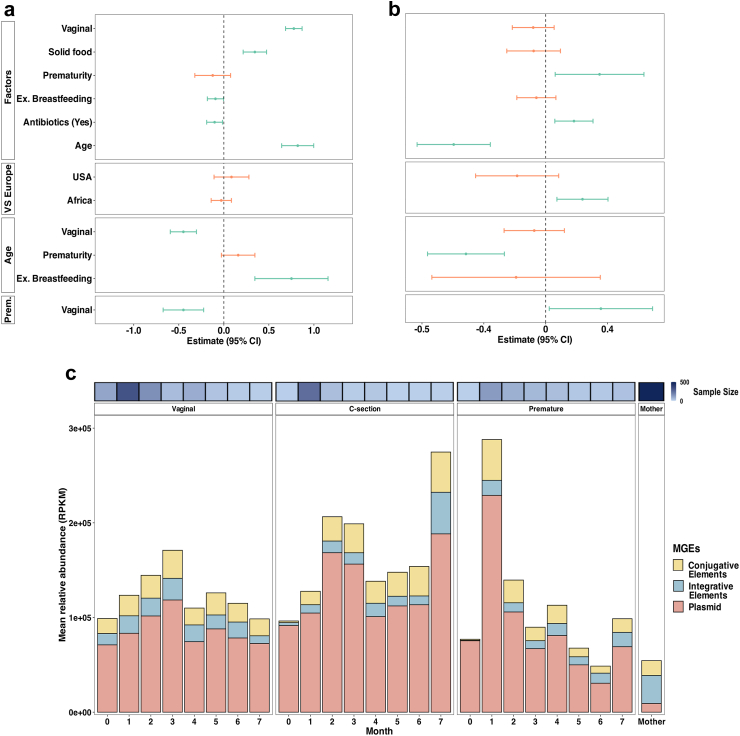
**Infant gut mobilome dynamics.** The impact of selected variables on the infant gut mobilome α-diversity (a) and abundance (b) as determined by linear mixed effect modelling (LMM) (*n* = 3497 samples from 1270 infants). The fixed effects used in LMM included infant age, the presence/absence of bead-beating, and the use of various sequencing platforms. The green error bars indicate statistically significant results (*p* < 0.05), while the orange error bars represent non-significant results (*p* > 0.05). (c) The relative abundance in reads per kilobase per million mapped reads (RPKM) of three mobile genetic element classes detected in the metagenomic data of infant and maternal samples. The ‘C-section’ (*n* = 330 samples from 173 infants) and ‘Vaginal’ groups (*n* = 750 samples from 513 infants) cover samples from full-term infants that were exclusively breastfed the first three months of life and not exposed to antibiotics during the displayed period. The ‘Premature’ group (*n* = 372 samples from 135 infants) covers samples from preterm infants without discrimination of birth mode, feeding, or exposure to antibiotics. In addition, 484 maternal samples were used in the analysis.

For the mobilome analysis, we used the MobileOG database^30^ in our reference-based pipeline and evaluated the impact of early-life factors on gut mobilome α-diversity (Fig. 2a) and relative abundance (Fig. 2b). MGE α-diversity (Shannon index) was higher in vaginally delivered infants, while exclusive breastfeeding and antibiotic use decreased MGE diversity. In contrast, both antibiotic exposure and prematurity were positively associated with MGE relative abundance. The association of prematurity with MGE abundance diminished with age, and infants from Southern African cohorts had higher MGE abundance compared to European cohorts. β-diversity analysis showed distinct monthly shifts in the mobilome (PERMANOVA test, adonis2, Bray–Curtis; *p* < 0.001, 999 permutations), with high inter-individual variability in younger neonates that converged to a more similar MGE composition over time (Figure S5b). Similarly to the resistome, over time, MGE diversity increased, and relative abundance decreased (Figure S6e and f, respectively; LMM; *p* < 0.01).

Compared to maternal samples, all infant groups showed a higher relative abundance of MGEs, with the highest levels in the ‘C-section’ and ‘Premature’ groups (Fig. 2c, Kruskal–Wallis followed by Dunn's test; mean adj. *p* = 0.002). Additionally, the distribution of MGE types varied between infants and adults. Plasmids were predominant in the infants' gut mobilome (median plasmid abundance at 39,818 RPKM in infants and 2834 RPKM in mothers, Wilcoxon rank-sum test; *p* < 0.01), whereas integrative elements were more common in mothers (median abundance at 11,551 RPKM in infants and 27,831 RPKM in mothers, Wilcoxon rank-sum test; *p* < 0.01).

### Bacterial species with known pathogenic potential carry the majority of ARGs

The observed differences in the gut resistome and mobilome across the three selected infant groups were likely influenced by variations in bacterial microbiota (Figure S7). We therefore investigated the interaction between infant gut microbiota, resistome, and mobilome composition. We found a moderate correlation between the α-diversity (Shannon index) of species-level microbiota composition, resistome, and mobilome (Figure S8a, Spearman's rho: 0.45, 0.44, and 0.23; *p*-values <0.01). Procrustes analysis further confirmed significant correlations between microbiota, resistome, and mobilome profiles, with correlation coefficients of 0.41, 0.46, and 0.53 (Figure S8b, Monte Carlo permutation; *p*-values <0.01, permutation 999).

**Fig. 3:**
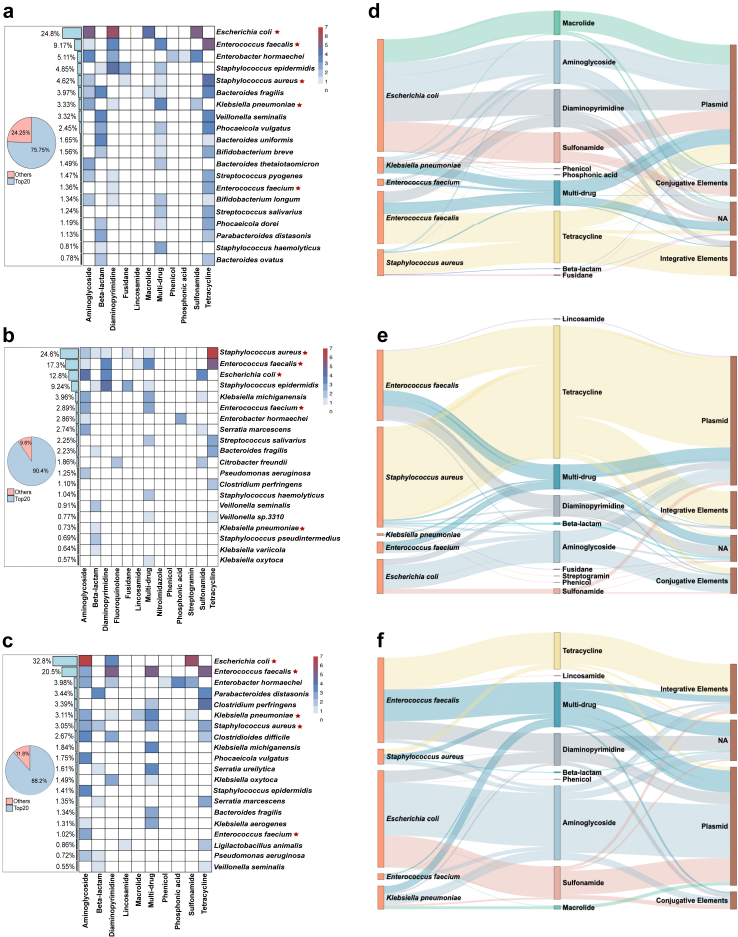
**Major bacterial hosts of infant gut resistome.** The heatmap shows the prevalence of antibiotic resistant gene (ARG) families in (a) ‘Vaginal’, (b) ‘C-section’, and (c) ‘Premature’ infant groups for the top 20 bacterial species associated with ARGs. The scale represents the log_2_ of the ARG count. Red stars in the heatmaps denote five bacterial species, which are significant contributors to global mortality, and these are also shown in Sankey diagrams of panels d–f. Each Sankey diagram connects indicated bacterial species to ARG families and mobile genetic element categories for samples from (d) ‘Vaginal’, (e) ‘C-section’, and (f) ‘Premature’ infant groups. The length of each species node signifies the total count of resistance genes, mobile genetic elements, or contigs assigned to a specific bacterium within each group, enabling intra-group analysis but not direct comparison between the infant groups (*i.e.,* the node lengths are not comparable across d, e, and f panels). The ‘C-section’ (*n* = 330 samples from 173 infants) and ‘Vaginal’ groups (*n* = 750 samples from 513 infants) are full-term infants that were exclusively breastfed the first three months of life and not exposed to antibiotics. The ‘Premature’ group (*n* = 372 samples from 135 infants) is preterm babies without discrimination of birth mode, feeding, or exposure to antibiotics. NA denotes contigs that have been assigned to both species and resistance genes but not to mobile genetic elements. Multi-drug is ARG conferring resistance to two or more antibiotic classes.

Using an assembly-based approach, we identified that the top 20 bacterial species carried 75.8%, 90.4%, and 88.2% of the detected ARGs (Table S8) for the ‘Vaginal’, ‘C-section’, and ‘Premature’ groups, respectively (Fig. 3). In the ‘Vaginal’ and ‘Premature’ groups, *E. coli* was the primary reservoir for ARGs, representing 24.8% and 32.8% of the detected ARGs, respectively. In the ‘C-section’ group, *E. coli* was the third most prevalent host, harbouring 12.8%. Fig. 3 shows pooled data from samples collected over the first seven months of life to capture overall ARG carriage patterns, while Figure S9 shows the tendencies of ARG carriage over time. Maternal samples had the smallest proportion of ARGs occurring on *E. coli-*annotated contigs, accounting for 8.5% of detected ARGs (Figure S10). Conversely, *Bacteroides uniformis* was the most prevalent host in adult samples, carrying 12.6% of detected ARGs. *E. faecalis* was the second most predominant host for ARGs in the ‘Vaginal’, ‘C-section’, and ‘Premature’ groups, contributing 9.17%, 17.3%, and 20.5% to the total ARGs, respectively.

We also examined the co-localisation of MGEs and ARGs in five bacterial species (*E. coli, Enterococcus faecalis, Staphylococcus aureus, Klebsiella pneumoniae, and Enterococcus faecium)* that are key contributors to global AMR-related mortality.^1^ Plasmids were the dominant MGEs co-localising with ARGs in all infant groups, with 55.1% in the ‘Vaginal’ group, 58.3% in the ‘C-section’ group, and 51.8% in the ‘Premature’ group (Figure S10c). In contrast, the maternal gut microbiota showed 38.4% ARGs co-localised with conjugative elements, 27.6% with plasmids, and 27.2% without MGEs on the same contig.

Despite our filtering step for clinically relevant ARGs, some of the detected ARGs can be endogenous genes that may not have an impact on responses to antibiotic treatment in cases of bacterial infection. We, therefore, checked for ARGs most relevant to antibiotic treatment failures, such as carbapenem and vancomycin resistance genes. Several CTX genes coding for extended-spectrum β-lactamases were present among the ARGs detected by the reference-based approach (Table S6), which, however, does not provide direct linkage to bacterial species. From the assembly-based approach, which yielded a smaller number of detected ARGs because of the quality requirements for short-read assembly (Table S8), we were able to link several important ARGs and know bacterial pathobionts. For example, a penicillin-binding protein conferring methicillin resistance in *Staphylococcus aureus* (*mecA*) was present on contigs annotated as belonging to *S. aureus* and other *Staphylococcus* species (Table S9). Further, several genes conferring resistance to aminoglycosides were linked to *Campylobacter jejuni* (*aph*) and *E. coli* (*npmA*). We also observed *qnr* and *bla* DHA genes, which confer resistance to fluoroquinolone and AmpC β-lactamase, respectively, in *Citrobacter freundii*, highlighting that commensal bacterial species act as a reservoir of clinically important ARGs.

We followed up on the observation of plasmid dominance in the infant gut mobilome by determining the link to the resistome. Using negative binomial regression (NB), we found that the count of ARGs co-localising with plasmids was significantly higher than with other MGE classes in infants during the first seven months (NB followed by Benjamini-Hochberg; mean adj. *p* < 0.01; Table S10). In maternal samples, integrative elements co-localised with more ARGs than other MGEs, but the difference was not statistically significant (*p* > 0.05). Comparing plasmid abundance and ARG co-localisation, mothers had significantly lower levels of plasmid sequences detected via the reference-based approach as well as those co-localising with ARGs on the same contig than infants (Figure S11, Kruskal–Wallis followed by Dunn's test; adj. *p* < 0.01; NB followed by Benjamini-Hochberg; mean adj. *p* < 0.01).

### Verification of bacterial hosts of ARGs by alternative approaches

To confirm the ARG-carrying bacterial taxa, we adopted an alternative analytical strategy besides the assembly-based approach. Precisely, we explored the association between identified ARGs and their bacterial carriers by clustering the short read-based taxonomic profiles into distinct microbiota community types (MCs) using non-negative matrix factorisation.^41^ Importantly, the purpose of the MC analysis was to identify the primary hosts of ARGs and defined community types, not to use MCs as a proxy of age-related microbiome development as in earlier studies.^49^^,^^50^

**Fig. 4:**
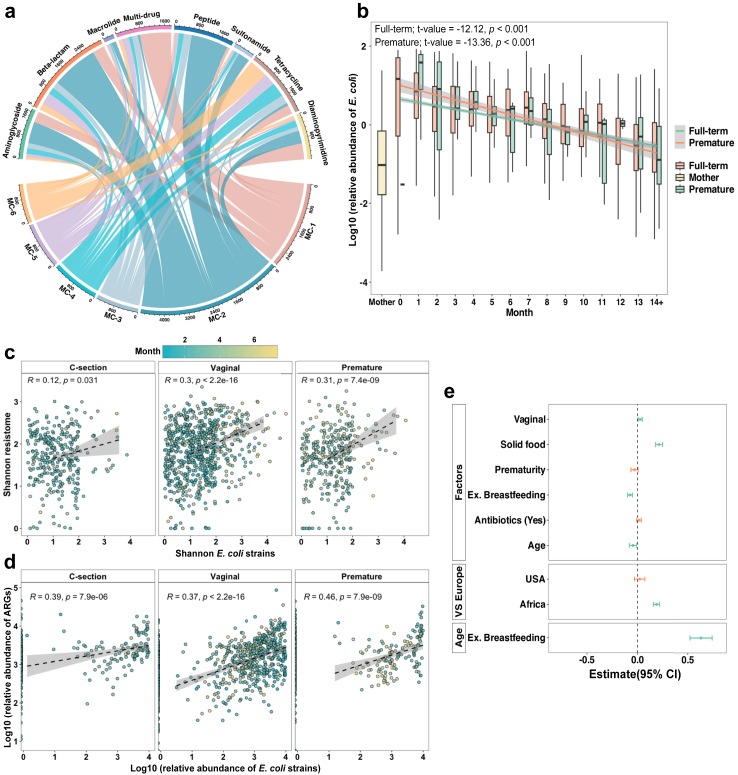
***E. coli* as a major host of antibiotic resistome.** (a) The relative abundance of each antibiotic resistant gene (ARG) family, measured in reads per kilobase per million mapped reads (RPKM), and predicted associations with six microbiota community (MC) types identified through Non-negative Matrix Factorisation (*n* = 3497 samples from 1270 infants). (b) A comparison of *E. coli* relative abundance between infants (*n* = 3497 samples from 1270 infants) and mothers (*n* = 484). The trajectory of *E. coli* abundance over time was analysed using linear mixed-effects modelling (LMM). The association between the α-diversity of *E. coli* strains and ARGs (c) and the relative abundance of *E. coli* strains and ARGs (d) in three selected infant groups was determined by Spearman's correlation. (e) The impact of selected variables on *E. coli* strains α-diversity employing LMM (*n* = 3497 samples from 1270 infants). The fixed effects used in LMM included infant age, presence/absence of bead-beating, and the use of various sequencing platforms. The green colour indicates a significant association, and the estimates are in a Confidence Interval (CI) of 95% as determined by LMM. The ‘C-section’ (*n* = 330 samples from 173 infants) and ‘Vaginal’ groups (*n* = 750 samples from 513 infants) are full-term infants that were exclusively breastfed the first three months of life and not exposed to antibiotics during the displayed period. The ‘Premature’ group (*n* = 372 samples from 135 infants) is preterm babies without discrimination of birth mode, feeding, or exposure to antibiotics. The green error bars indicate statistically significant results (*p* < 0.05), while the orange error bars represent non-significant results (*p* > 0.05).

We identified six distinct MCs (Figure S12) and subsequently aligned the ARGs against the MC types. This analysis revealed that MC2, dominated by the *Escherichia* genus, harboured the highest relative abundance of the resistome (Kruskal–Wallis followed by Dunn's test; adj. *p* < 0.01; Fig. 4a). MC1, characterised by a high relative abundance of *Enterococcus*, was associated with the second-highest resistome relative abundance.

We also considered that *E. coli* could have been identified as a major host of ARGs in our assembly-based analysis due to potential database overrepresentation, which might have resulted in a high number of contigs or ARGs assigned to *E. coli*. However, we did not identify a correlation between the number of contigs assigned to specific bacteria and their role as ARG hosts (Figure S13a). For instance, within the ‘C-section’ group, *E. coli* had the highest number of assigned contigs, yet it was the third most significant host for the total count of ARGs (Fig. 3c). We also checked the number of ARGs assigned to *E. coli* and other bacterial species in the CARD database. *E. coli* count was the fourth highest, after *Pseudomonas aeruginosa, Acinetobacter baumannii,* and *Klebsiella pneumonia* (Table S11).

Following these quality controls, we clustered samples according to *E. coli* relative abundance, employing mean and standard deviation for classification. Notably, samples with high *E. coli* relative levels showed a significantly higher ARGs relative abundance (Wilcoxon rank-sum test; *p*-value <0.01; Figure S13b). Additionally, upon adjusting the analyses for *E. coli* abundance employing LMM, we found that as the abundance of *E. coli* increased, the abundance of ARGs also increased significantly (LMM; t-value = 11.3, *p* < 0.001). When conducting the same analysis for *B. longum*, a bacterial host not commonly associated with ARG carriage,^4^ we detected an opposite trend (Wilcoxon rank-sum test; *p*-value <0.01; Figure S13c; LMM; t-value = −3.46, *p* < 0.001). Additionally, we found a notably weak correlation between *E. coli* and *B. longum* abundance (Spearman's rho: Vaginal = −0.08, C-section = 0.11, Premature = 0.15, Mother = 0.16; all *p*-values <0.05).

### *E. coli* strain-level dynamics influence the carriage of ARGs

Upon ascertaining *E. coli* as a dominant host for ARGs in the infants' gut and observing the trend of decreasing resistome and mobilome as infants age (Figure S6), we investigated whether this reduction is linked to a decrease in *E. coli* relative abundance over time. *E. coli* levels were significantly higher in infants compared to mothers (Kruskal–Wallis followed by Dunn's test; mean adj. *p* = 0.003) and showed a notable decrease in both full-term and premature infants as they aged, aligning closer to maternal levels (LMM; *p* < 0.001; Fig. 4b). Additionally, samples with high *E. coli* relative abundance exhibited significantly greater ARG and plasmid abundances (Wilcoxon rank-sum test; *p* < 0.01; Figures S13d). Adjusting for *E. coli* abundance using LMM, we found a significant increase in ARGs and plasmid abundance with higher *E. coli* levels (LMM; t = 11.3 and 26.3, respectively, *p* < 0.001). These trends suggest that the reduction in *E. coli* relative abundance over time is a key factor driving the decrease in both resistome and plasmid relative abundance as infants grow.

Finally, we employed StrainGE^32^ to explore *E. coli* strain-level dynamics, as differences in strain diversity could explain the higher *E. coli* relative abundance in infants compared to adults. We found a weak correlation between *E. coli* strains and ARGs α-diversities across the infant groups, with Spearman's mean R values of 0.24 (*p* < 0.001; Fig. 4c). The correlation was stronger in the ‘Vaginal’ and ‘Premature’ groups, where *E. coli* was the dominant ARG carrier during the first months of life (Figure S7). Additionally, we identified a moderate correlation between the relative abundance of *E. coli* strains and ARGs, with Spearman's mean R values around 0.40 (Fig. 4d).

An LMM analysis of *E. coli* strain α-diversity, based on the Shannon index, indicated higher strain diversity associated with vaginal birth, solid food introduction, and Southern African geographic location (Fig. 4e). Conversely, C-section delivery, prematurity, and European location were associated with lower *E. coli* strain diversity. Antibiotics did not significantly impact *E. coli* strain α-diversity, although there was a trend toward a positive effect. Notably, *E. coli* strain α-diversity decreased over time.

## Discussion

This meta-analysis comprehensively examined the dynamics of ARG and MGE carriage in the infant gut microbiota over time. We determined that vaginally delivered infants have higher ARG α-diversity but at lower cumulative relative abundance than those born via C-section, consistent with prior research.^8^ This finding likely reflects a more diverse bacterial population in vaginally delivered infants, which mainly originates from the maternal gut microbiota^51^ and may introduce a wider array of ARGs but at a controlled abundance. In contrast, premature infants displayed lower ARG diversity but higher ARG relative abundance compared to full-term babies, likely attributed to the lower bacterial diversity yet higher abundance of ARG carriers like *E. coli* and *Enterococcus faecalis*.^6^

We observed evident geographical variations, with two African cohorts showing higher resistome diversity and ARGs relative abundance compared to European cohorts, suggesting an impact of factors such as non-prescription antibiotic use, poor water quality and inadequate sanitation in low-resource settings, the occurrence of endemic antibiotic-resistant bacteria, and potential maternal colonisation with bacteria carrying ARGs.^2^^,^^52^ On the other hand, the US cohorts displayed a higher diversity of ARGs but at a lower abundance than the European ones. These geographical variations underscore the need for a global strategy to address the antibiotic resistance burden.

Our research did not find a significant impact of breastfeeding on the infants' gut resistome α-diversity and relative abundance, contrary to a previous study,^7^ possibly due to differences in the cohort type and size. Specifically, this study of stool samples from 46 preterm infants, collected in a cross-sectional design, used a generalised linear model to determine the impact of diet on ARG load.^7^ The results showed that formula exposure was correlated with a higher ARG burden. On the other hand, a characterisation of 4132 metagenomes from 963 infants in six countries did not show that breastfeeding alone has a significant effect on gut resistome.^53^ This study identified that although the feeding type was significantly associated with the overall resistance, formula feeding seemed to be a primary driver of a lower ARG load. It remains to be determined whether the effect of breastfeeding on ARG carriage might be modulated by gestational age and other perinatal factors.

Our study highlights *E. coli* and *E. faecalis* as primary reservoirs for ARGs in the infant gut, with co-localisation of their ARGs with plasmids. The latter predicts the role of plasmids in ARG dissemination. Moreover, the evidence for increased relative abundance of both MGEs and ARGs after antibiotic exposure strongly suggests that antibiotic use can act as a trigger for the transfer of resistance genes within the gut microbiota.

In line with recent studies,^54^^,^^55^ we also identified an association between a high *E. coli* abundance with elevated ARGs and plasmid relative abundance, suggesting that diminishing *E. coli* might reduce ARG levels in the infant gut.^54^ Yet, a complete displacement might not be advantageous as there are likely yet undiscovered ecological roles for *E. coli* in the infant gut. An alternative strategy is thus a replacement of resistant strains with those that provide health benefits and do not carry ARGs. However, replacing ARG-carrying strains might also be associated with risks because the new strains can be seeded by ARG-carrying mobile elements. Importantly, we noted a decline in *E. coli* diversity over time, likely reflecting the maturation and stabilisation of the microbiota as infants grow. The simultaneous longitudinal decline of ARG load might have critical consequences for reducing susceptibility to AMR infections.

Our meta-analysis also showed that the magnitude of β-diversity variance explained by some of the factors of interest (*e.g*., birth mode) was similar to the process-related variables such as the choice of sequencing platform. This is in line with previous reports.^56^^,^^57^ However, the influence of the process-related variables overlaps with the biological difference in the cohorts, as each cohort used a specific processing protocol. The process-related taxonomical variation is, therefore, only partially comparable with the variance explained by biological variables, such as birth mode, that are present in every cohort of the dataset. Despite the importance of this issue, only a few studies report the variance explained by process-related variables. For example, in a single large cohort of infants, where the process variations should be minimal, previous studies have reported that 8–15% of the variance could be explained by the technical variations,^58^^,^^59^ an effect size close to primary biological drivers of the microbiota composition.

We acknowledge several limitations of this study, notably the incomplete metadata concerning exposures, such as details on antibiotic use (the type, duration, for treatment of which infections, exact timing after birth, maternal intrapartum use, etc.). This may influence the interpretations and applicability of our findings, especially when it comes to differences between vaginally and C-section-delivered infants, with the mothers of the latter group getting frequent antibiotic prophylaxis. Moreover, a majority of premature infants included in our meta-analysis are from high-income countries with lower endemic resistance rates, and the findings thus may only partially reflect the gut resistomes of preterm infants from other regions. Finally, the heterogeneity of studies required manual data harmonisation, which might not have captured the population characteristics in sufficient resolution (*e.g*., assumption about solid food status).

Another limitation is that the commonly used metagenomic assembly tools can capture the ARG repertoire to some extent but do not completely encompass the diversity of genomic contexts present in a sample.^60^ For that reason, we combined an assembly-based approach with a reference-based approach to minimise potential biases while determining the overall carriage of ARGs and MGEs. One limitation of the reference-based approach is that bacterial hosts of ARGs and MGEs can only be predicted but not reliably determined. Therefore, we applied the assembly-based approach to identify the bacterial hosts. However, as earlier demonstrated,^60^ linking bacterial hosts to all potential ARGs and MGEs is currently not possible via the assembly-based approach. Similarly, with current assemblers, it is not possible to reconstruct the entire repertoire of plasmids from metagenomic data.^61^ We circumvented this bottleneck by identifying MGEs on annotated contigs and by mapping short reads to the mobileOG database.^30^ Still, we want to highlight that this approach does not provide the whole landscape of the gut mobilome.

It is also important to note that for many of the detected ARGs, there is insufficient evidence to demonstrate their role in providing resistance to commonly used antibiotics in the context of the gut microbiome. While colonisation by drug-resistant microorganisms has been associated with a higher risk of infection in individuals with weakened immune systems,^62^^,^^63^ additional research is needed to fully understand the implications of ARG-carrying bacteria within the gut microbiota. In this study, we focused on the natural history of ARG carriage and the influence of various perinatal exposures. However, many of the early life exposures can be difficult to modify, making them challenging targets for reducing ARG carriage. Lastly, our study has been solely based on computational analyses, and the predictions regarding the relationships between bacterial hosts, ARGs, and MGEs need to be replicated and confirmed in experimental models.

Despite these limitations, our meta-analysis deepens the current understanding of how early-life factors impact gut microbiota, resistome, mobilome, as well as *E. coli* strain dynamics. This research is crucial for strategies aiming to mitigate AMR.

## Contributors

VKP conceived the study and secured the funding. VKP performed the literature search, and AB, HN, AJP, AK, and VKP systematically screened studies and applied inclusion and exclusion criteria established by all authors. The individual metadata curation was handled by AB, HN, AJP, CJ, and AK, with AJP overseeing the integration and final curation of datasets. These authors have also accessed and verified the underlying data. AB crafted the bioinformatics pipeline and ran the analyses, while AJP provided specialised support and identified key confounding variables for the downstream analyses. AB also performed statistical and machine learning analyses, data visualisation, and drafted the initial manuscript. AB supplied AJP with SLURM scripts for reference- and assembly-based approaches to convert them into a Snakemake workflow. AB created a Snakemake workflow for the pre-processing steps. The types of data analyses were decided upon by consensus among all authors. VKP supervised data analyses and manuscript preparation. All authors provided critical feedback on the manuscript and approved the final version for submission.

## Data sharing statement

All shotgun metagenomic datasets^6^^,^^8^^,^^11^, ^12^, ^13^, ^14^, ^15^, ^16^, ^17^, ^18^, ^19^, ^20^, ^21^, ^22^ are publicly available. Metadata, including all accession numbers for the included samples and the BioProject IDs of the datasets, are available in (Table S2), along with the variable dictionary (Table S3). The identified marker families from CARD database (v.3.2.7) and MobileOG database (v.1.6) are available here (https://doi.org/10.5281/zenodo.11491353). The complete code for the pipelines is available at: https://github.com/Ahmedbargheet/Snakemake_short_reads_preprocessing/, https://github.com/aponsero/Resistome_ReadBased_Snakemake/and https://github.com/aponsero/Resistome_AssemblyBased_Snakemake.

## Declaration of interests

AJP has received a consulting fee from the University of Tampere unrelated to this manuscript. All other authors declare no competing interests.

## Appendix A Supplementary data

Supplementary Tables

Supplementary info
