# Supplementary material for: Dynamics of gut resistome and mobilome in early life: a meta-analysis
Source: eBioMedicine. 2025 Mar 5;114:105630. doi: 10.1016/j.ebiom.2025.105630 (PMC11929092; doi:10.1016/j.ebiom.2025.105630)
Supplement: Supplementary info [file mmc2.docx]

Supplementary Information for

**Dynamics of gut resistome and mobilome in early life:**

**A meta-analysis**

Bargheet et al.

**CONTENT**

**Supplementary Figures**

**Figure S1** Prisma flow diagram of selected articles and search words used in the literature search.

**Figure S2** Distribution of % unclassified reads per study**.**

**Figure S3** Overview of the metadata.

**Figure S4.** Variables importance and heterogeneity among cohorts.

**Figure S5** PCoA of resistome and mobilome compositions.

**Figure S6** General gut resistome and mobilome dynamics in full-term infants.

**Figure S7** The relative abundance of bacterial genera.

**Figure S8** Spearman’s correlation and Procrustes analysis of metagenomic profiles.

**Figure S9** The number of unique antibiotic resistance genes (ARGs) carried by five bacterial species.

**Figure S10** Bacterial host of resistome and mobilome detected in maternal samples.

**Figure S11** Comparison of the plasmid counts across the study groups.

**Figure S12** Clustering the gut microbiota by community types.

**Figure S13** Relative abundance of resistome identified in the samples with high and low abundance of *E. coli* and *B. longum*.

**Supplementary Tables**

**Table S1** Summary of the studies included in the meta-analysis.

**Table S5** PERMANOVA models performed on infant samples.

**Table S12** Top 10 bacterial species in the CARD database

**
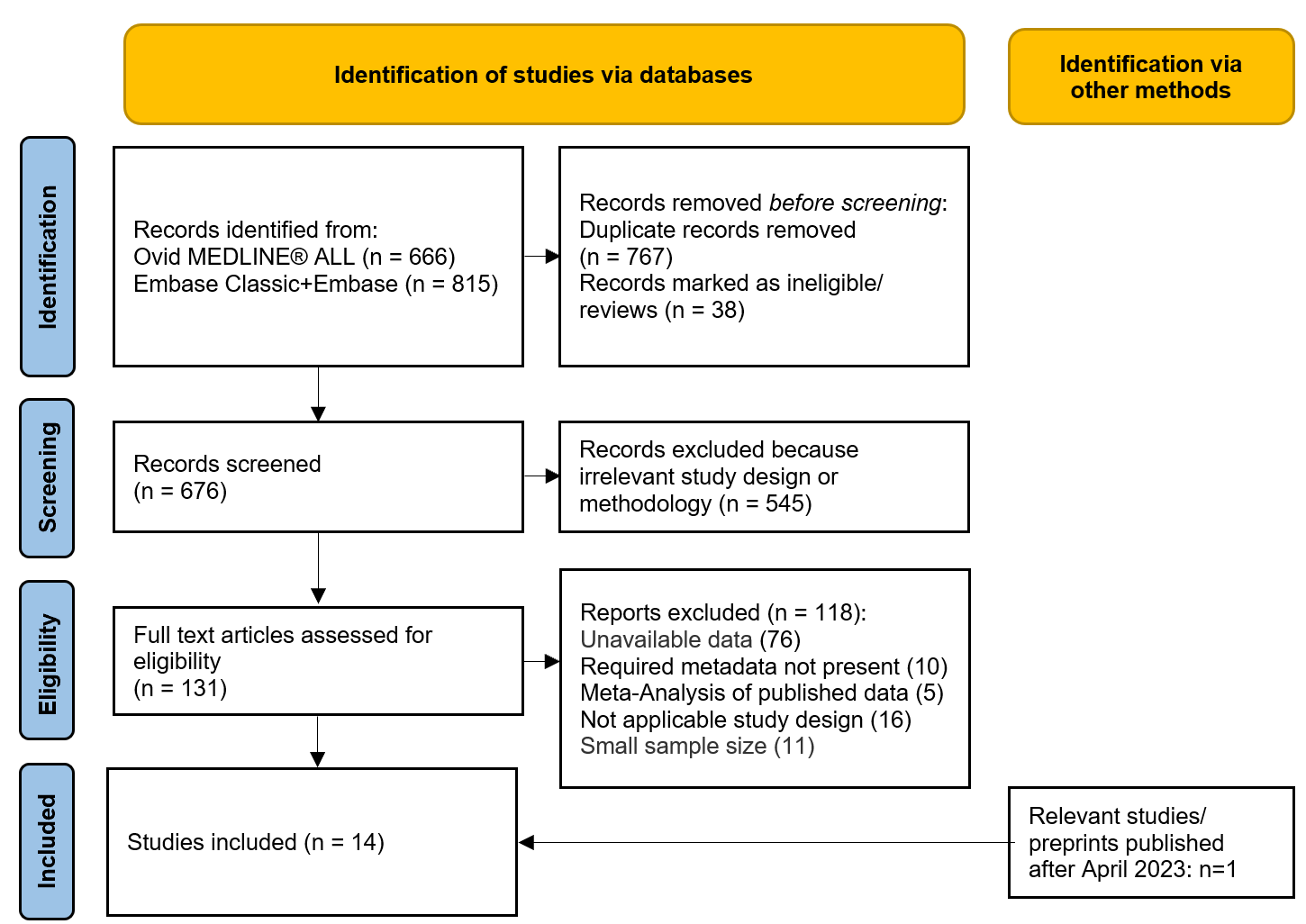
Figure S1. A) Prisma flow diagram. B) Search words used in the databases.** The words in each box, including the Mesh terms, were combined with «OR», and the search results based on the terms in each box were combined with «AND».


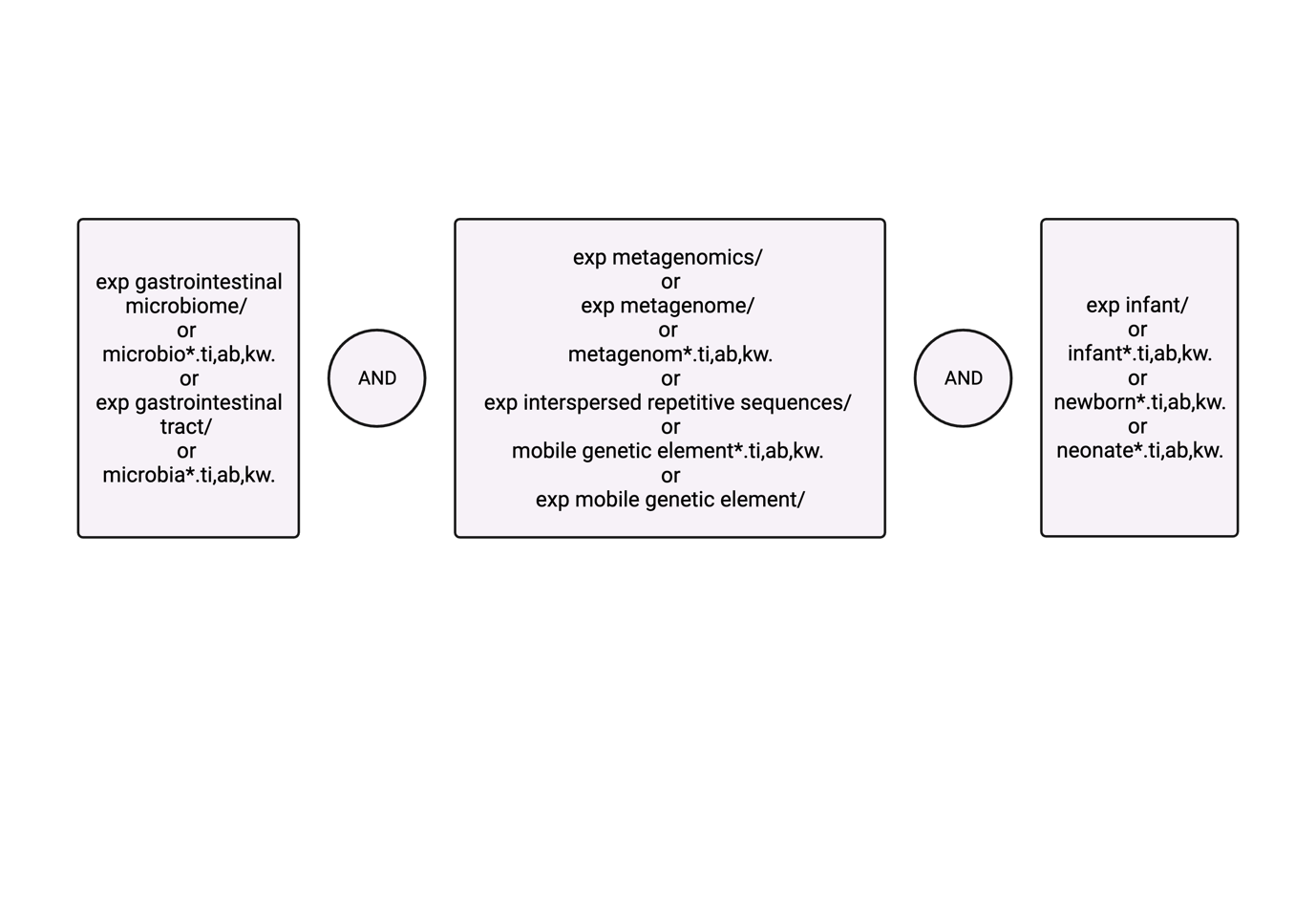


**b**

**a**


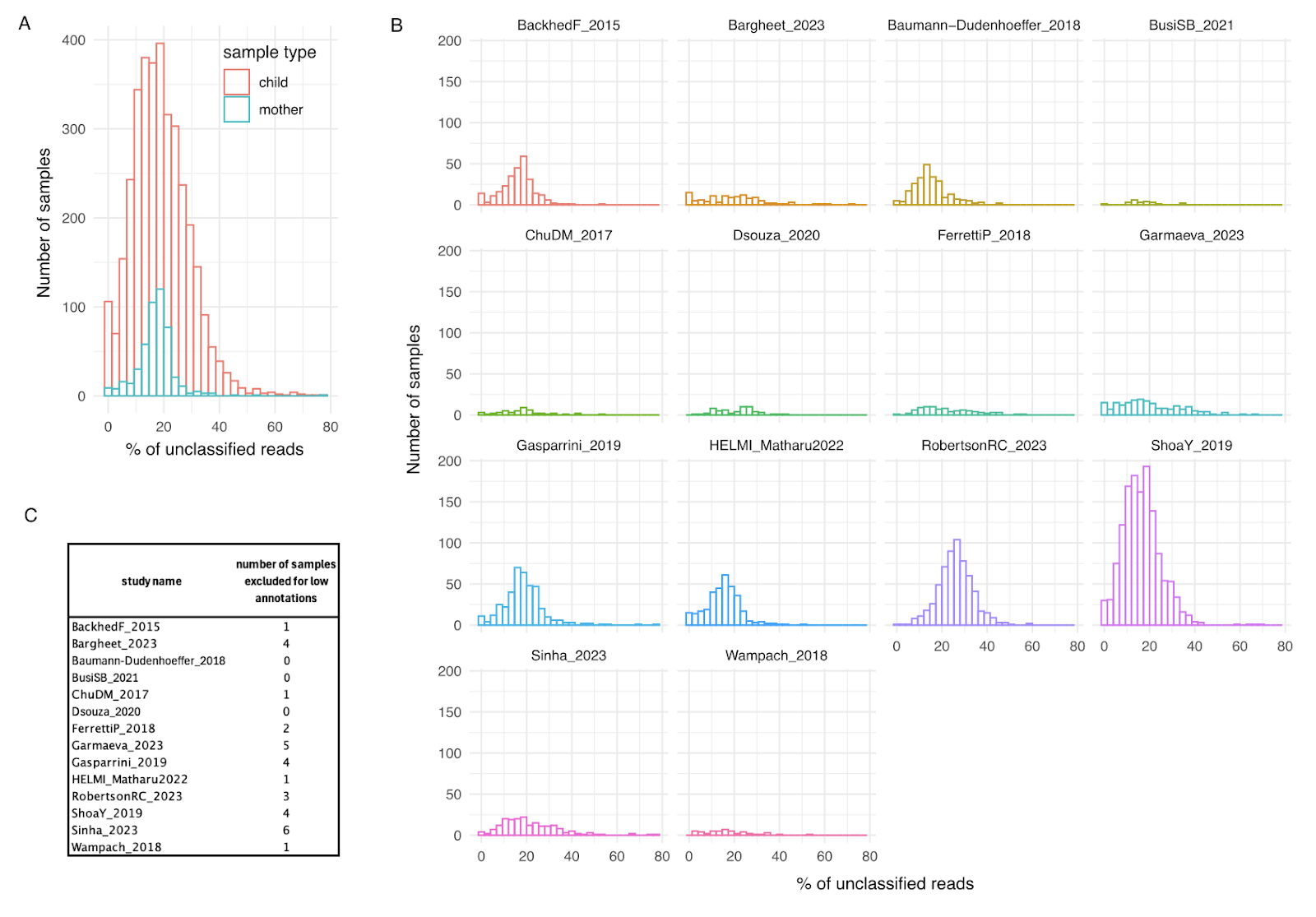


**a**

**b**

**c**

**Figure S2. Distribution of % unclassified reads per study.** a) Proportion of unclassified reads in the total dataset for the different sample types. b) Distribution of the proportion of unclassified reads in the different cohorts. c) Number of samples excluded per cohort based on the number of unclassified reads.


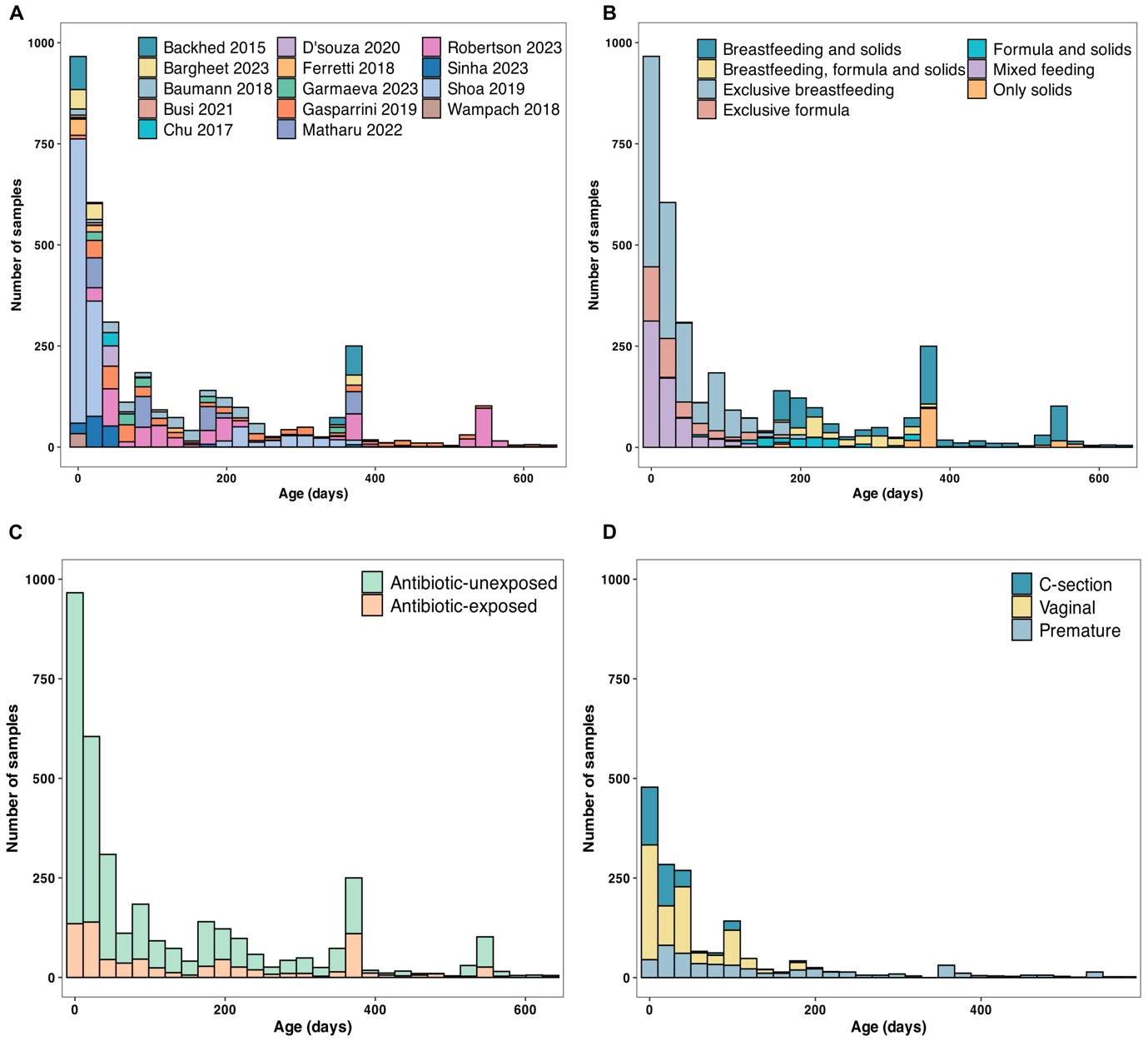


**a**

**b**

**c**

**d**

**Figure S3. Overview of the metadata.** Distribution of 3981 samples used in the meta-analysis according to a) individual cohorts, b) feeding practices, and c) antibiotic exposures at the time of sample collection for the infant samples. D) Three selected infant groups were used to illustrate the differential development of gut microbiome, mobilome, and resistome. Labels for three representative infant groups: ‘C-section’ – infants born by C-section delivery at term, breastfed the first three months of life, and not exposed to antibiotics (n = 133 with 330 samples); ‘Vaginal’ - infants born by vaginal delivery at term, breastfed the first three months of life, and not exposed to antibiotics (n = 384 with 750 samples); ‘Premature’ - infants born either by C-section or vaginal delivery before term, mixed fed, and often exposed to antibiotics (n = 83 with 372 samples).

**
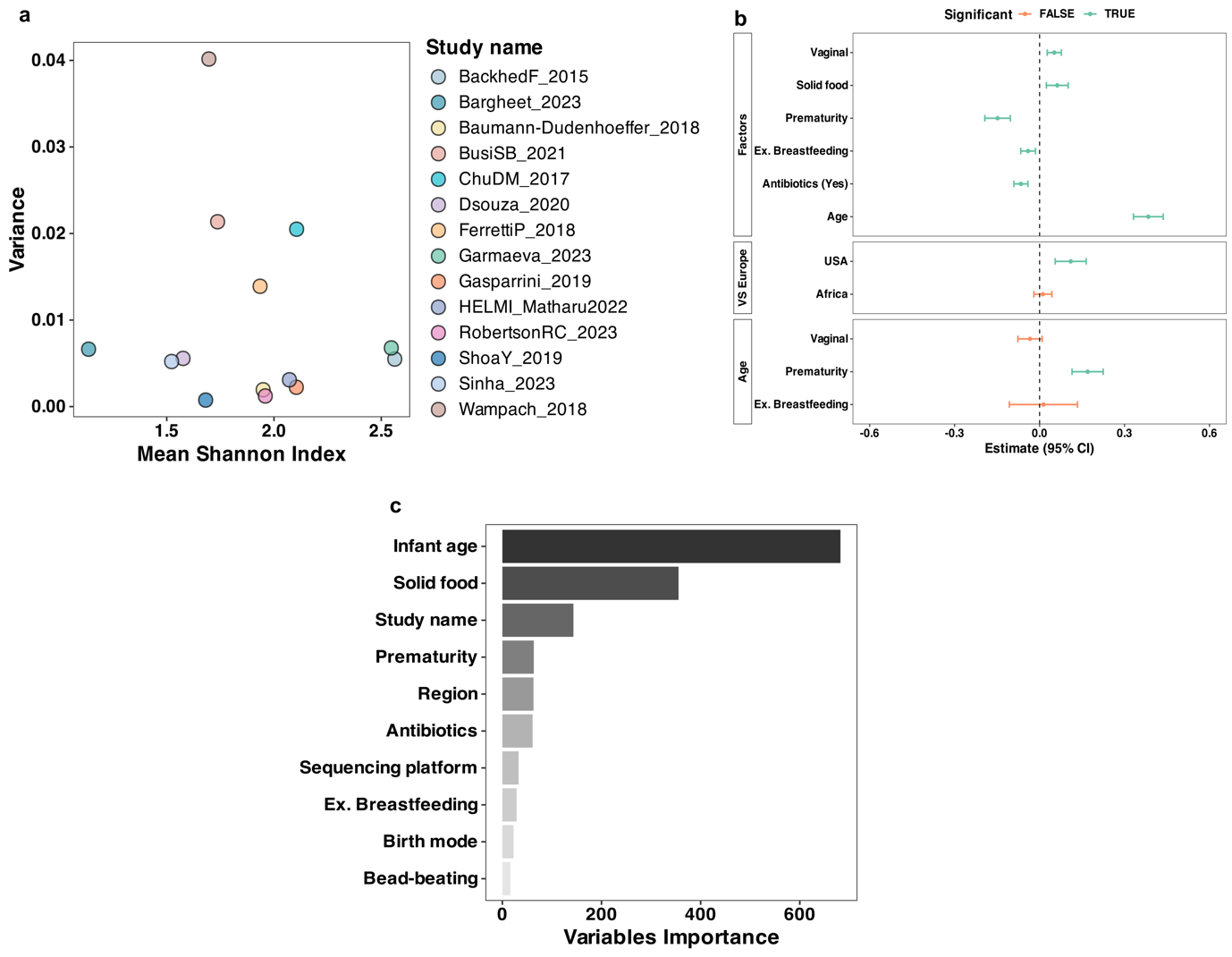
Figure S4. Variables importance and inter-cohort heterogeneity.** a) The variability in bacterial diversity (mean Shannon index) across cohorts. The *I^2^* statistic was used to estimate the proportion of variability in microbial diversity attributable to between-study heterogeneity rather than chance. The analysis revealed substantial heterogeneity among cohorts (*Tau^2^* = 0.15, SE = 0.059, *I^2^*=97.81%, *p* = 0.0001). Each dot represents a study, and its position in the plot depends on the variance and the mean Shannon index values. Dots that are close together (both in terms of mean Shannon index and variance) suggest those studies have similar bacterial diversity and variability within their samples. Dots that are farther apart, especially in variance (y-axis), indicate that these studies differ in their diversity patterns or consistency. b) The impact of selected variables on the infant gut microbiota α-diversity as determined by a linear mixed effect modelling (LMM). The fixed effects used in LMM included infant age, the presence/absence of bead-beating, and the use of various sequencing platforms. The green error bars indicate statistically significant results, while the orange error bars represent non-significant results. c) The contribution of each predictor on the bacterial diversity (Shannon index) using the *vip* R package upon running a random forest.


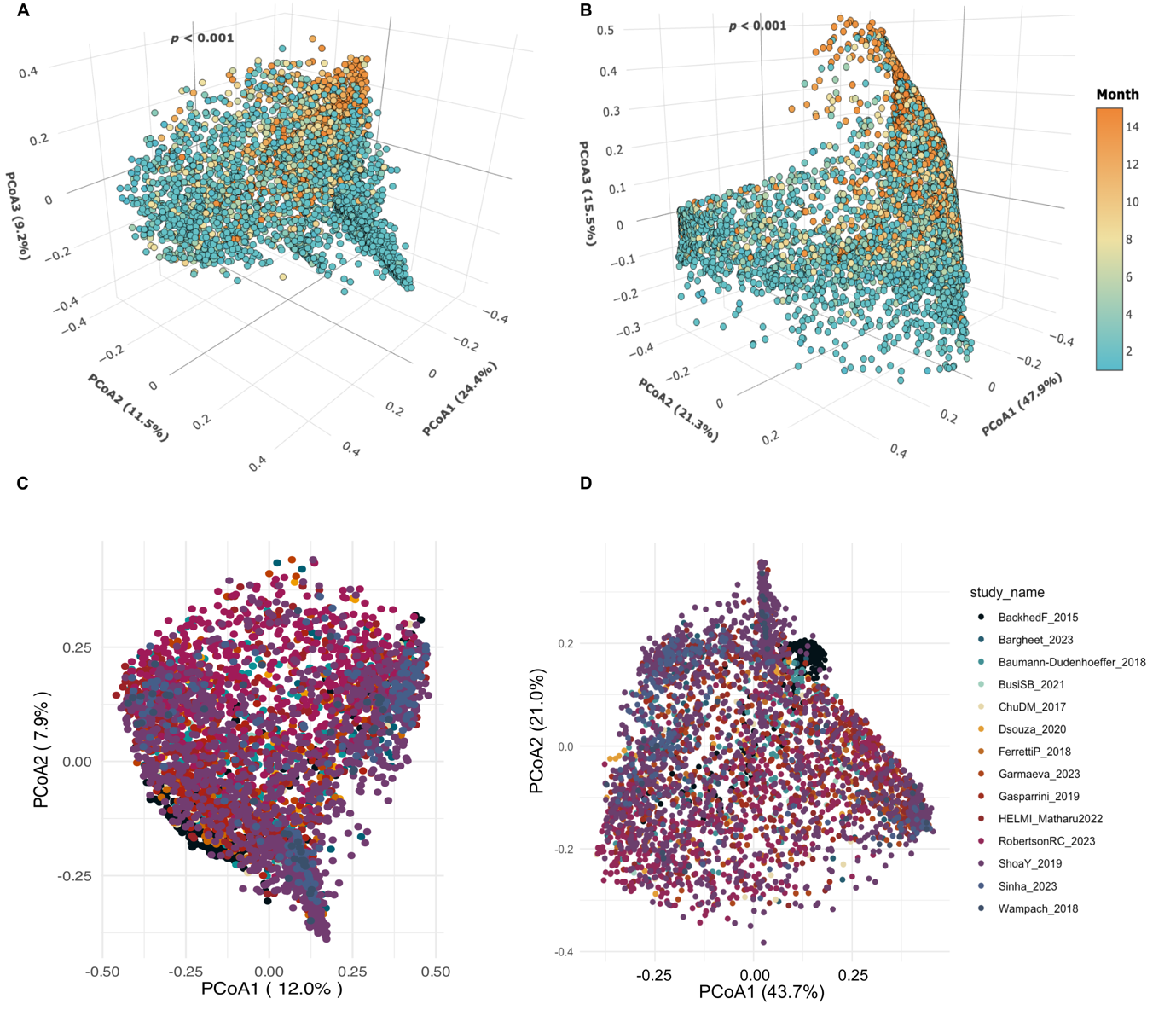


**a**

**c**

**d**

**b**

**Figure S5.** **Principal Coordinate Analysis (PCoA)** describing the samples’ resistome (a), mobilome (b) across ages, using Bray-Curtis distance. Description of the resistome (c) and mobilome (d) per cohort. The p-value was calculated by adonis2 with premutation 999.

***
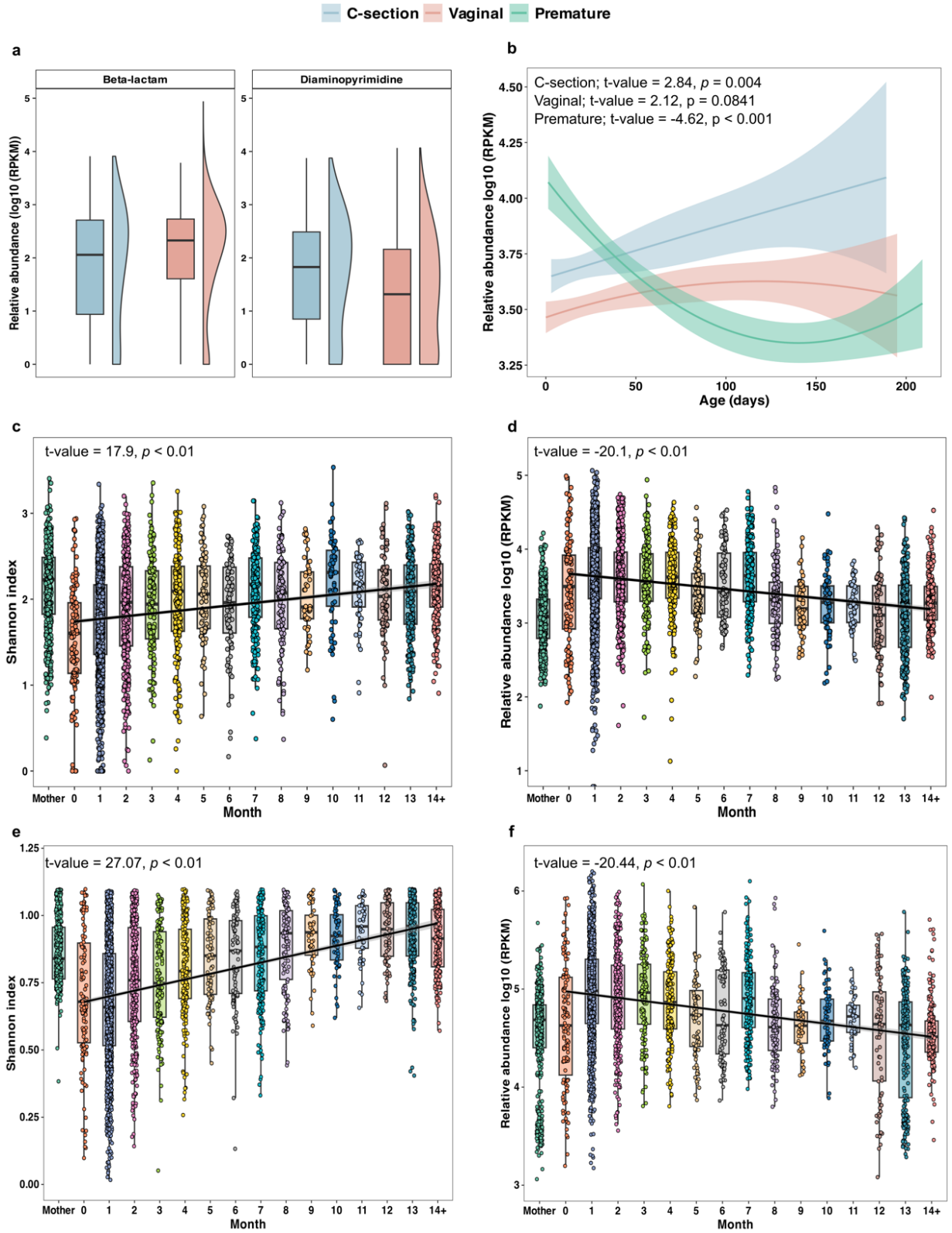
***

**Figure S6. General dynamics of the gut resistome and mobilome in full-term infants.** (a) Two antibiotic classes showed significantly differential abundance (Benjamini-Hochberg adjusted p < 0.01) in the samples of full-term infants delivered either vaginally or by C-section (without discrimination of feeding type or exposure to antibiotics) in the period of 0-3 months. The ANCOM-BC model was adjusted for sampling age, using 2077 samples in the analysis (n=1090). (b) Log_10_ of ARG relative abundance trend across three infant groups (see groups descriptions in the main text). (c) Resistome α-diversity trends, estimated using the Shannon index. (d) Log_10_ of ARG relative abundance quantified in reads per kilobase per million mapped reads (RPKM). (e) The trend of mobilome α-diversity as estimated by the Shannon index. (f) Log_10_ of MGE relative abundance in RPKM. Each data point represents an individual sample, and p and t-values were estimated with a linear mixed effect model (LMM). Analyses in panels b-e were performed on 2985 samples of 1131 full-term infants.

**
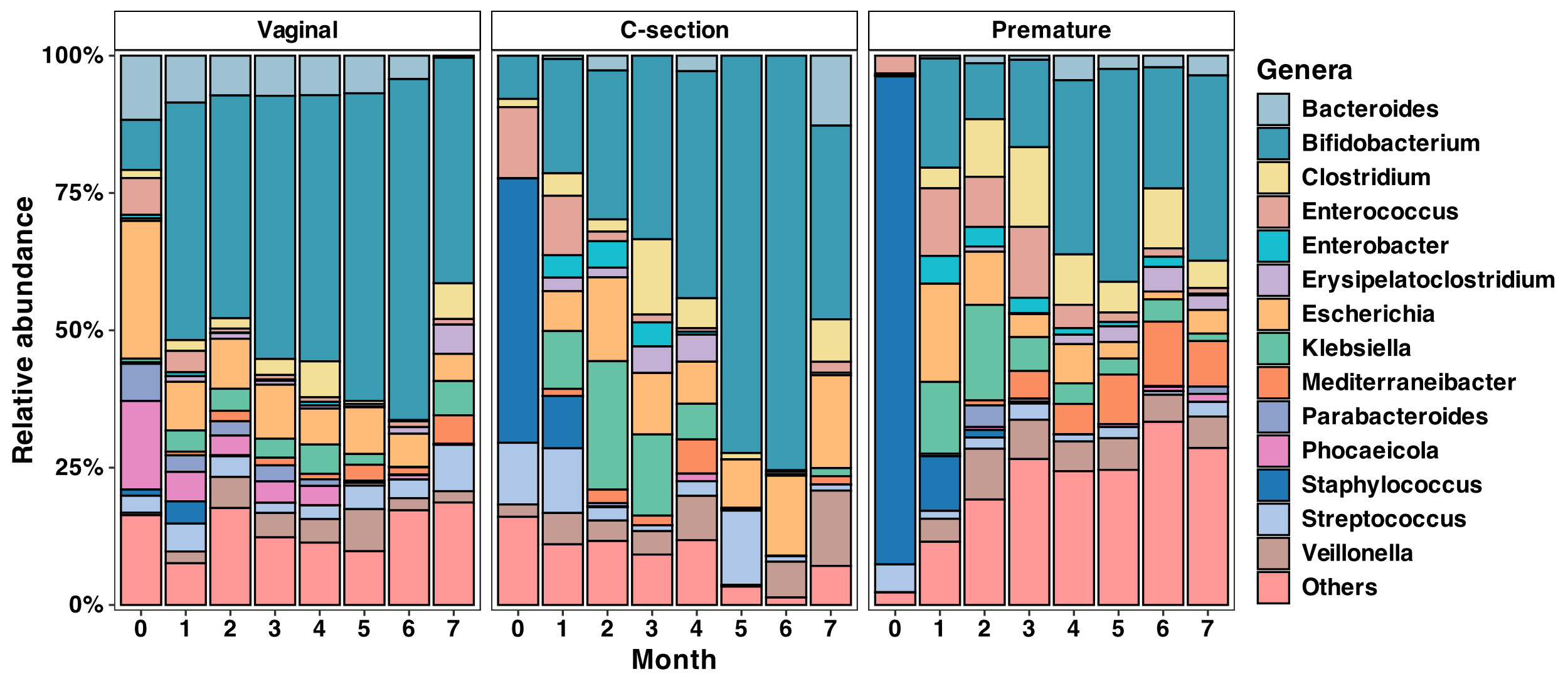
Figure S7. The relative abundance of bacterial genera.** Genera with abundance (< 1%) were merged into the “Others” category. The ‘C-section’ and ‘Vaginal’ groups cover samples from full-term infants that were exclusively breastfed the first three months of life and not exposed to antibiotics during the displayed period. The ‘Premature’ group covers samples from preterm infants without discrimination of birth mode, feeding, or exposure to antibiotics.

**
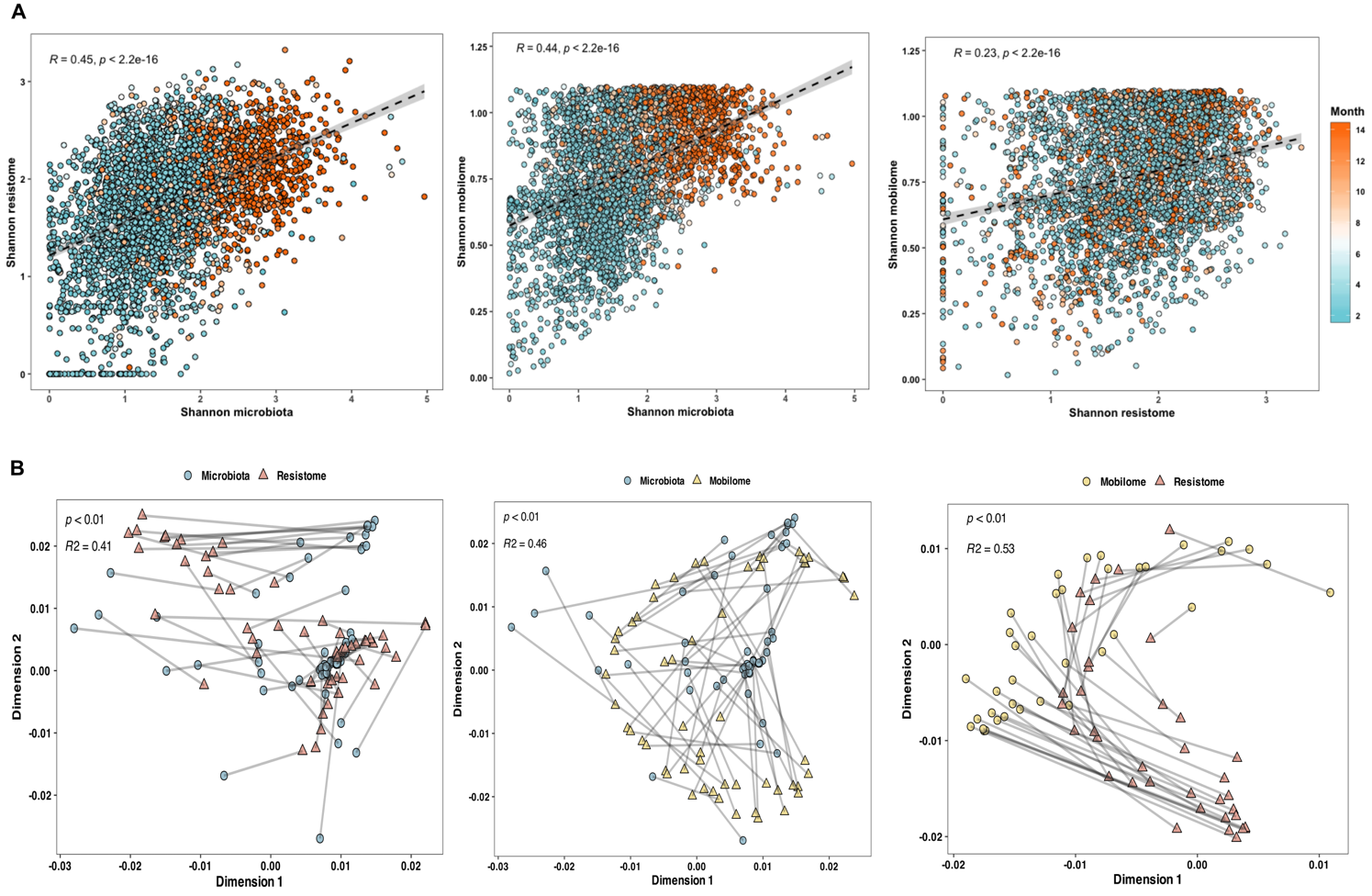
Figure S8. Spearman’s correlation and Procrustes analysis of three metagenomic profiles.** (a) The correlation between the α-diversity of bacterial microbiota, resistome, and mobilome was determined by Spearman’s correlation analysis. (b) The R^2^ coefficients describe the correlation between the composition of microbiota, resistome, and mobilome, as determined by the Procrustes analysis, including all infant samples. For the visualisation, we used only samples from 0 to 3 months in the figures. The *p*-values were estimated by the Monte Carlo permutation test; 999 permutations, and including all samples in the meta-analysis.

**b**

**a**

**
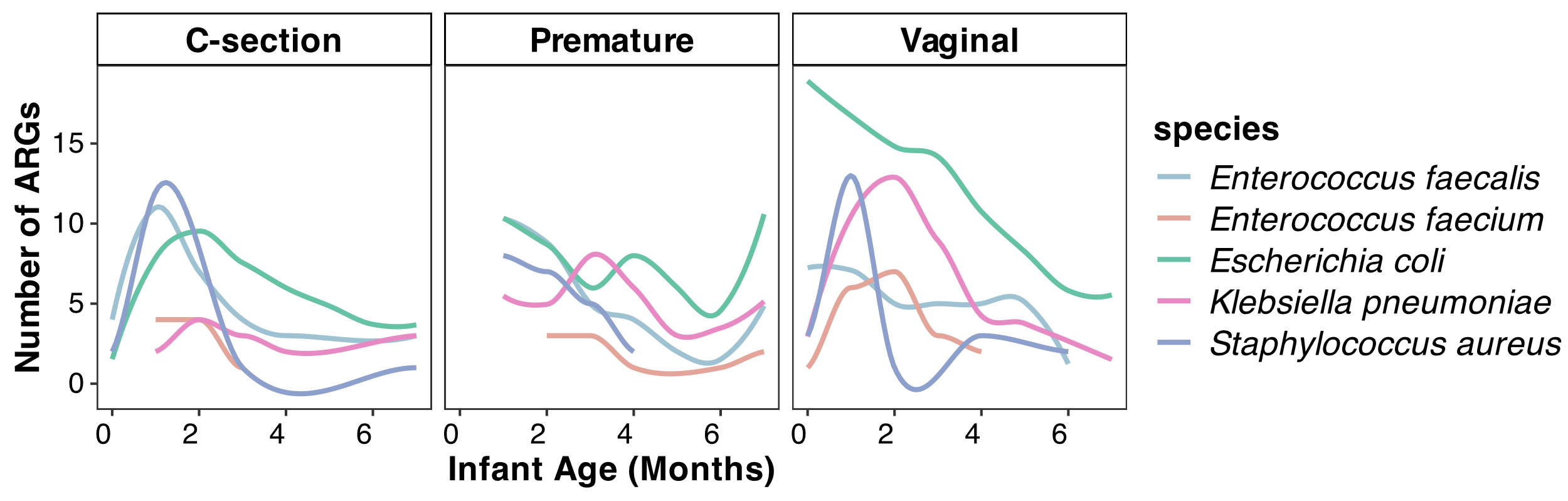
Figure S9. The number of unique antibiotic resistance genes (ARGs) carried by five bacterial species** present in the infant gut. Each line represents a bacterial species, with trend lines fitted using LOESS smoothing to highlight changes over time. The ‘C-section’ and ‘Vaginal’ groups cover samples from full-term infants that were exclusively breastfed the first three months of life and not exposed to antibiotics during the displayed period. The ‘Premature’ group covers samples from preterm infants without discrimination of birth mode, feeding, or exposure to antibiotics.


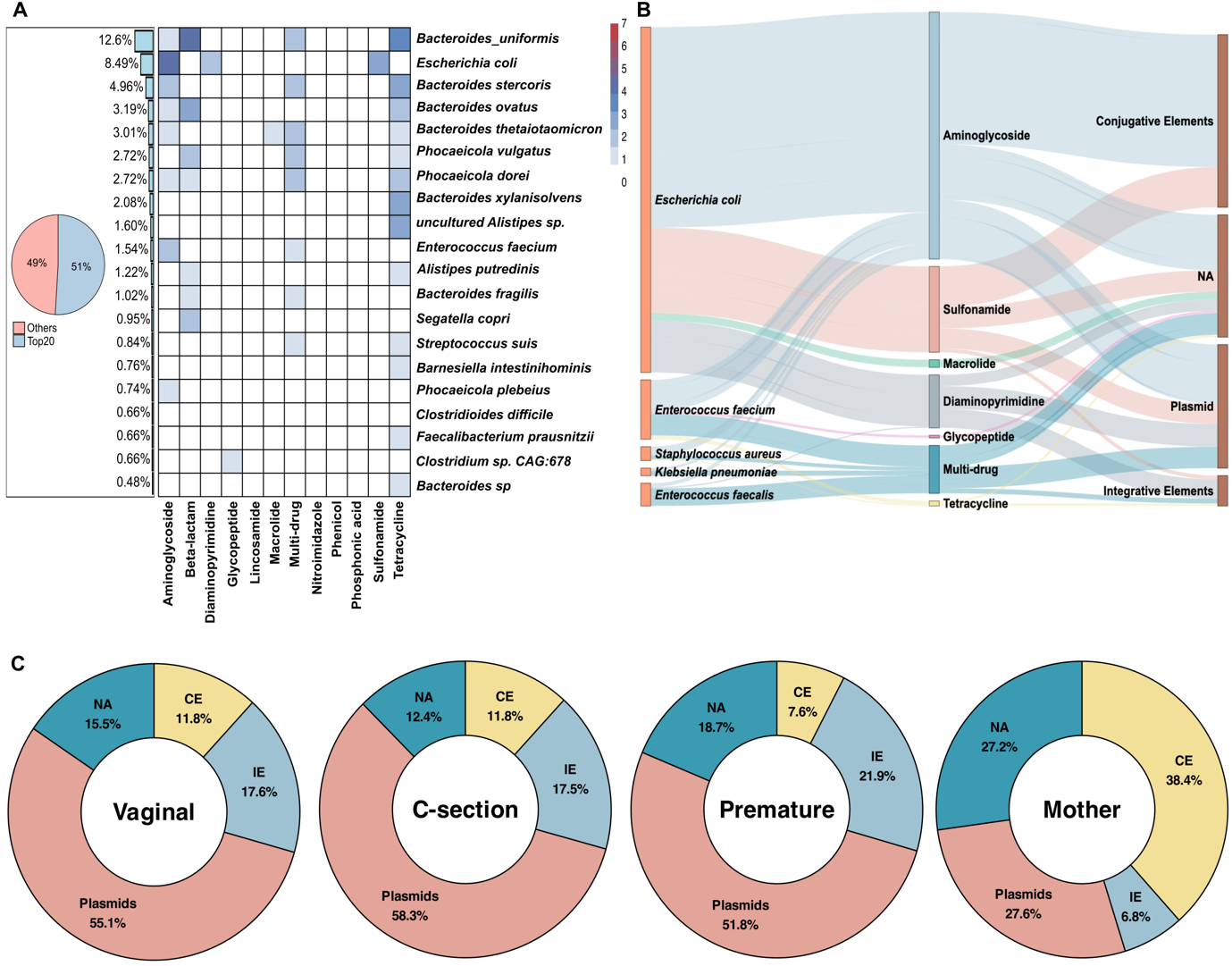


**a**

**b**

**c**

**Figure S10:** (a) The prevalence of ARG families of the top 20 bacterial species in the maternal gut microbiota. The heatmap scale represents the log_2_ of the ARGs count. (b) Sankey diagram illustrating the connection in maternal samples between resistance genes, mobile genetic elements, and selected five bacterial species with known pathogenic potential, which are major contributors to global mortality. The length of each species node signifies the total count of resistance genes. (c) The pie charts illustrate the percentages of ARGs identified as co-localized with the top five pathogenic bacterial species in conjunction with different mobile genetic elements throughout the study. The ‘C-section’ and ‘Vaginal’ groups are full-term infants that were exclusively breastfed the first three months of life and not exposed to antibiotics during the observed period. The ‘Premature’ group are preterm babies without discrimination of birth mode, feeding, or exposure to antibiotics. IE and CE denote integrative elements and conjugative elements, respectively. NA denotes contigs that have been assigned to both species and resistance genes but not to mobile genetic elements.


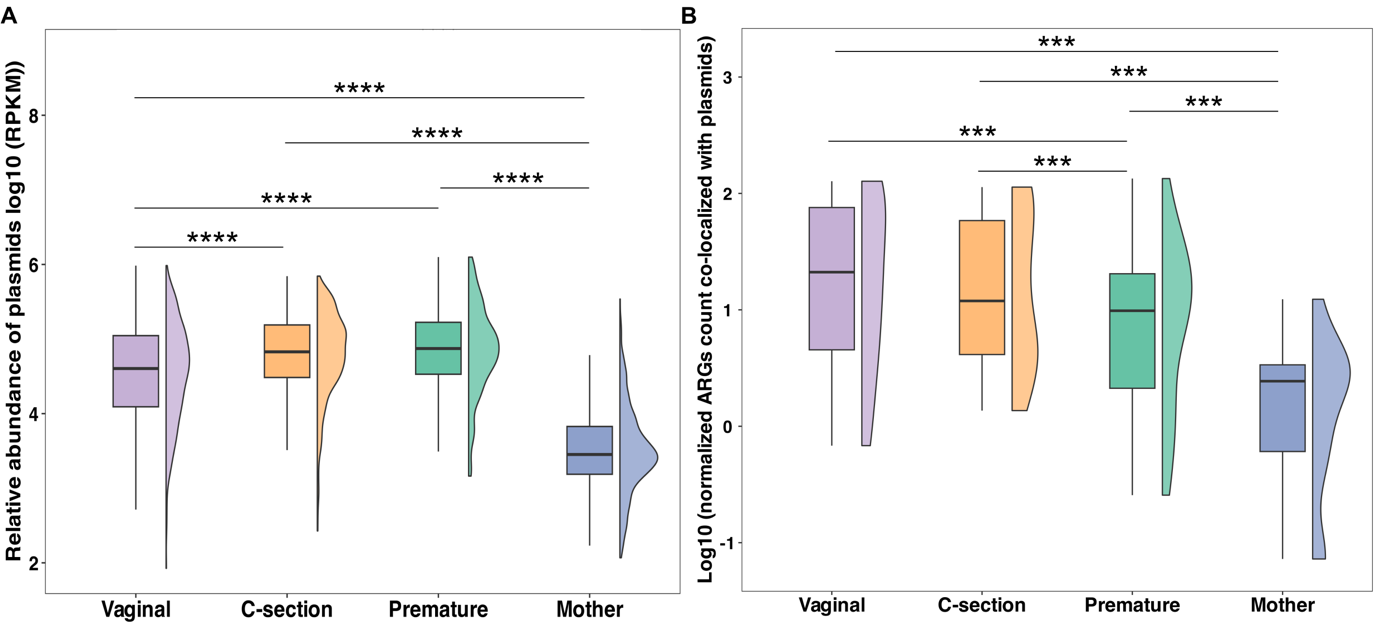


**a**

**b**

**Figure S11:** **Comparison of the plasmid counts across the study groups**. a) Comparison of the plasmid relative abundance, determined by the reference-based approach in reads per kilobase reference per million mapped reads (RPKM), across the study groups. The *p* values were computed using a Kruskal-Wallis followed by Dunn’s test (**** adj. *p*-value < 0.001). b) Co-localization of the ARGs and plasmids across the study groups, as determined by the assembly-based approach. The normalization was performed by dividing the count of the ARG or the MGE by the library size for each sample and multiplying this by one million. The distributions are displayed to the right of the boxplots. The *p* values were computed using a negative binomial regression (*** *p*-value < 0.001, ** *p*-value < 0.01). The ‘C-section’ and ‘Vaginal’ groups are full-term infants that were exclusively breastfed the first three months of life and not exposed to antibiotics during the observed period. The ‘Premature’ group are preterm babies without discrimination of birth mode, feeding, or exposure to antibiotics.


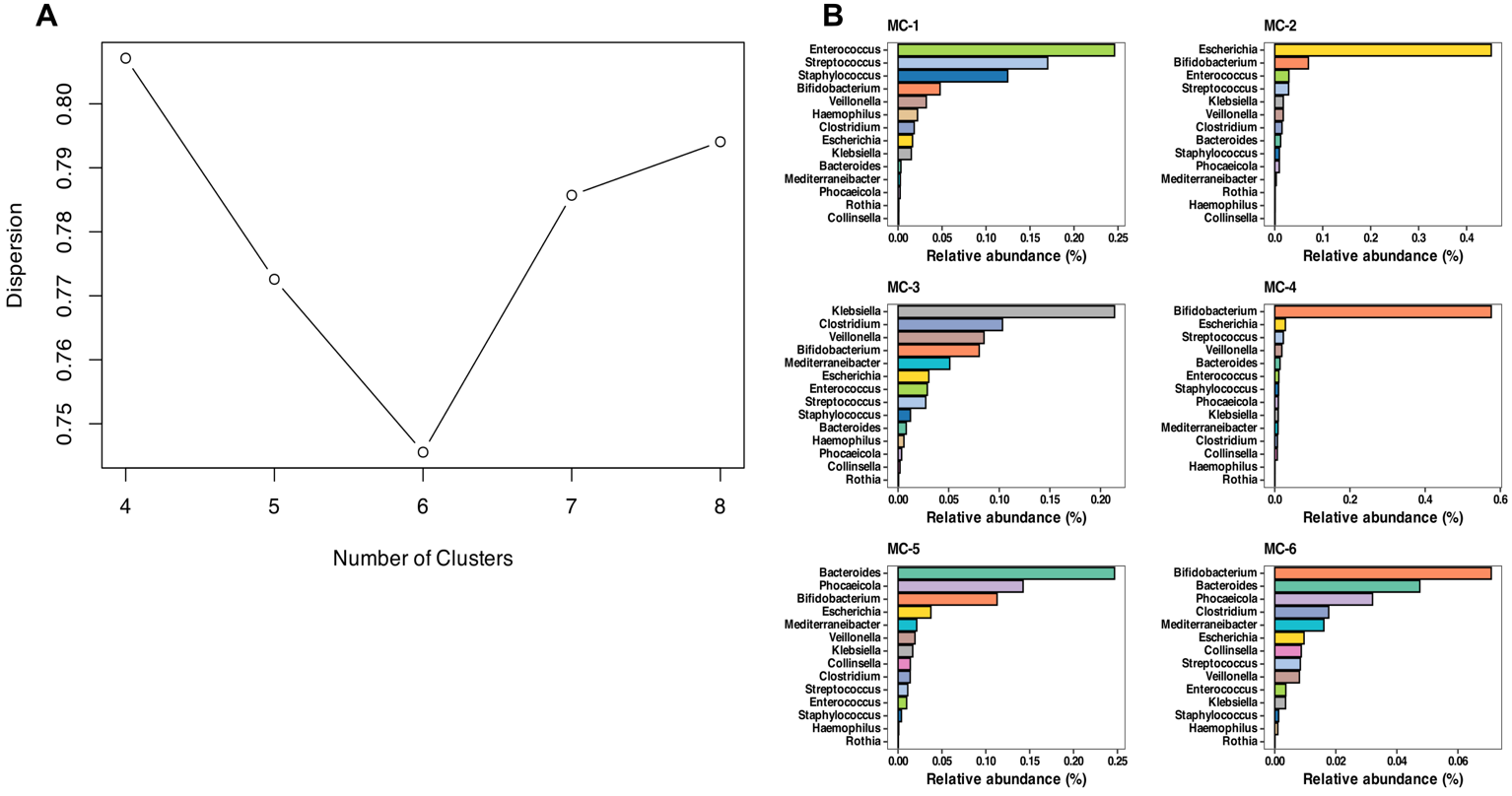


**a**

**b**

**Figure S12:** **Clustering the gut microbiota by community types**. (a) Determination of the most stable and consistent clusters across the samples using (the Brunet method and number of runs = 8 by Non-negative Matrix Factorization (NMF). (b) The composition of each microbiota community type (MCs), as identified through NMF.


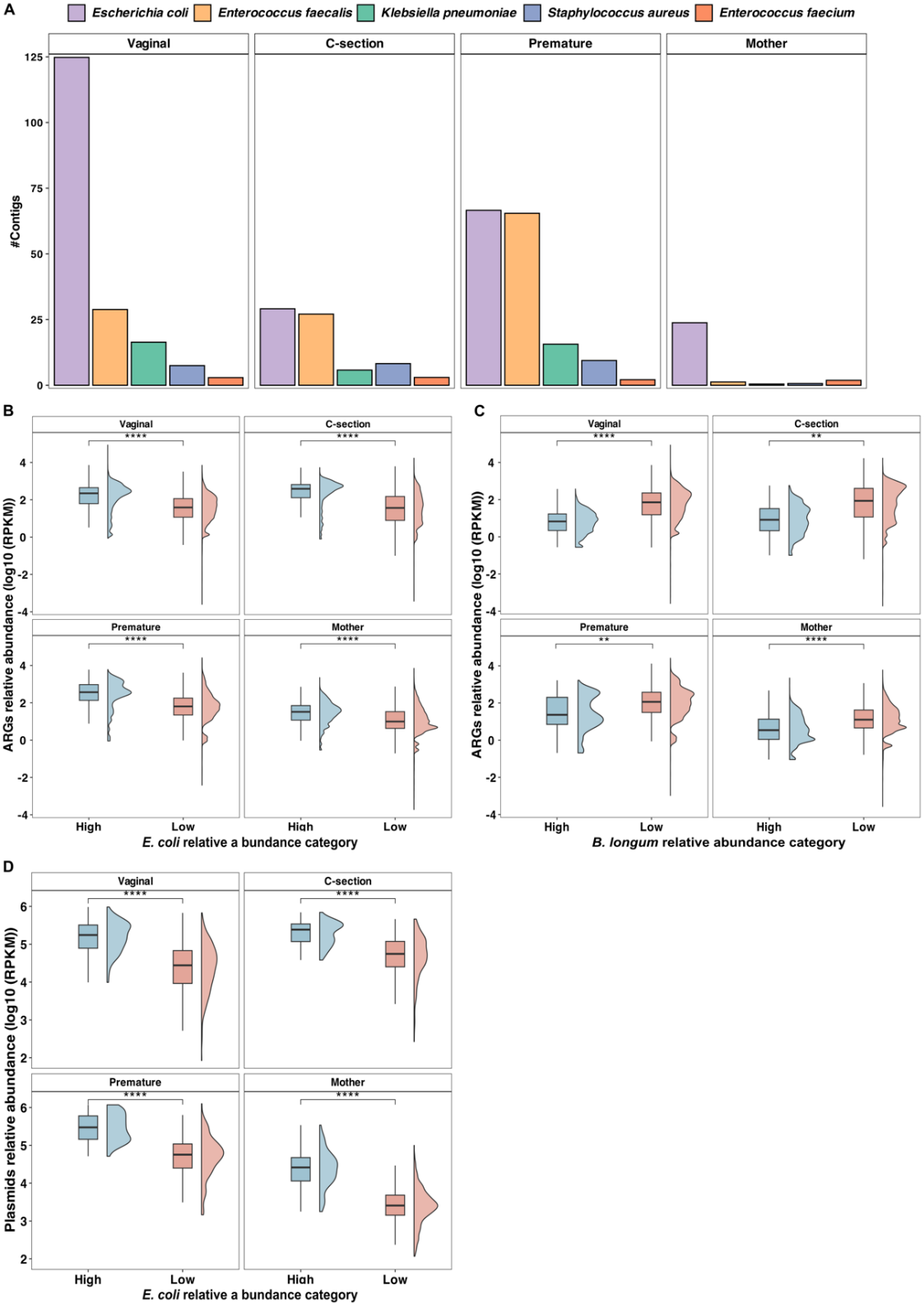


**a**

**b**

**c**

**d**

**Figure S13** **Relative abundance of resistome identified in the samples with high and low abundance of E. coli and B. longum**. (a) The total count of contigs assigned to the top five pathogenic bacterial species that are major contributors to global mortality. The identified relative abundance of resistome in reads per kilobase per million mapped reads (RPKM) in the samples with high and low abundance of (b) E. coli and (c) Bifidobacterium longum. (d) The identified relative abundance of plasmids RPKM in the samples with high and low abundance of E. coli. The distributions are displayed to the right of the boxplots. The p values were computed using the Wilcoxon rank-sum test (*** p-value < 0.001). The ‘C-section’ and ‘Vaginal’ groups are full-term infants that were exclusively breastfed the first three months of life and not exposed to antibiotics during the observed period. The ‘Premature’ group are preterm babies without discrimination of birth mode, feeding, or exposure to antibiotics.

**Table S**1**:** Summary of the studies. Abbreviations: FT: Full-term, PT: Preterm, C: C-section, V: Vaginal, F: Female, M: Male

| **Study name** | **#Infants** | **BioProject ID** | **#Infant samples** | **#Mothers** | **#Mothers sample** | **#Samples** | **#Term** | **#Born method** | **#Infant with abx** | **#Execlusivly breastfed** | **Country** | **Continent** | **#Sex** | **Total reads** | **Average number of reads/ sample** | **Standard deviation number of reads/ sample** |
| --- | --- | --- | --- | --- | --- | --- | --- | --- | --- | --- | --- | --- | --- | --- | --- | --- |
| Backhed  2015 | 95 | ERP005989 | 180 | 94 | 94 | 274 | FT:95 | C:15 V:80 | No:86 Yes:09 | No:31 Yes:64 | SWE | Europe | F:53 M:42 | 1,762,047,165 | 18,547,865 | 3,890,938 |
| Bargheet  2023 | 69 | PRJNA898628 | 117 | ـــــــ | ـــــــ | 117 | FT:09 PT:60 | C:36 V:33 | No:17 Yes:52 | No:05 Yes:64 | NO | Europe | NA | 215,573,050 | 3,124,247 | 6,671,598 |
| Baumann-Dudenhoeffer  2018 | 31 | PRJNA473126 | 244 | ـــــــ | ـــــــ | 244 | FT:19 PT:12 | C:17 V:14 | No:27 Yes:04 | No:25 Yes:06 | USA | North America | F:16 M:15 | 7,713,427 | 3,856,714 | 321,517 |
| Busi  2021 | 6 | PRJNA595749 | 23 | ـــــــ | ـــــــ | 23 | FT:06  PT:0 | C:02 V:04 | No:6. Yes:0 | No:03 Yes:03 | LUX | Europe | F:3 M:3 | 103,023,361 | 17,170,560 | 8,173,714 |
| ChuDM  2017 | 35 | SRP078001 | 36 | 11 | 11 | 47 | FT:33 PT:02 | C:08 V:27 | No:31 Yes:4 | No:18 Yes:17 | USA | North America | F:19 M:16 | 651,286,764 | 18,608,193 | 5,392,201 |
| Dsouza  2020 | 56 | PRJNA549787 | 59 | ـــــــ | ـــــــ | 59 | FT:56  PT:0 | C:12 V:44 | No:55 Yes:01 | No:0  Yes:56 | ZAF | Africa | F:25 M:31 | 137,592,055 | 4,438,453 | 1,439,722 |
| Ferretti  2018 | 24 | PRJNA352475 | 67 | 21 | 21 | 88 | FT:24  PT:0 | C:0 V:24 | No:23 Yes:01 | No:0  Yes:24 | ITA | Europe | NA | 501,520,998 | 20,896,708 | 38,226,376 |
| Garmaeva  2024 | 28 | NA | 99 | 29 | 98 | 197 | FT:28  PT:0 | C:03 V:25 | No:28 Yes:0 | No:12 Yes:16 | NL | Europe | F:15 M:14 | 361,291,463 | 12,903,267 | 2,566,774 |
| Gasparrini  2019 | 55 | PRJNA489090 | 395 | ـــــــ | ـــــــ | 395 | FT:17 PT:38 | C:44 V:11 | No:17 Yes:38 | No:54 Yes:01 | USA | North America | F:23 M:32 | 270,373,916 | 4,915,889 | 1,851,842 |
| Matharu  2022 | 78 | PRJEB52774 | 279 | 50 | 50 | 329 | FT:78  PT:0 | C:23 V:55 | No:72 Yes:06 | No:05 Yes:73 | FIN | Europe | F:38 M:40 | 1,522,770,358 | 19,522,697 | 6,572,484 |
| Robertson  2023 | 251 | PRJEB51728 | 606 | ـــــــ | ـــــــ | 606 | FT:224 PT:27 | C:13 V:238 | No:199 Yes:52 | No:140 Yes:111 | ZWE | Africa | F:112 M:139 | 1,436,787,259 | 5,770,230 | 1,797,998 |
| Shoa  2019 | 499 | ERP115334 & ERP024601 | 1192 | 174 | 174 | 1366 | FT:449  PT:0 | C:245 V:254 | No:422 Yes:77 | No:283 Yes:216 | GBR | Europe | F:240 M:257 NA:2 | 4,757,142,208 | 9,533,351 | 2,053,735 |
| Sinha  2023 | 28 | NA | 167 | 22 | 22 | 189 | FT:28  PT:0 | C:28 V:0 | No:28 Yes:0 | No:24 Yes:04 | NL | Europe | F:17 M:11 | 390,776,397 | 13,956,300 | 4,177,620 |
| Wampach  2018 | 15 | PRJNA379120 | 33 | 14 | 14 | 47 | FT:15  PT:0 | C:08 V:07 | No:15 Yes:0 | No:06 Yes:09 | LUX | Europe | F: 5 M:10 | 236,658,911 | 15,777,261 | 5,002,301 |

**Table S5 PERMANOVA models performed on infant samples***

(adonis2 PERMANOVA test on Bray-Curtis beta-diversity distances, number of permutations: 999, marginal effect model)

**A: Including all samples**

1. Impact of a specific study

*Equation: dist ~* Sequencing depth *+ sample age group + study name*

|  | Df | Sum Of Sqs | R2 | F | Pr(>F) |
| --- | --- | --- | --- | --- | --- |
| Sequencing depth | 1 | 0.66 | 0.00039 | 1.7778 | 0.013 |
| sample age group | 4 | 120.77 | 0.07091 | 81.6501 | 0.001 |
| study name | 13 | 76.23 | 0.04476 | 15.8571 | 0.001 |
|  |  |  |  |  |  |
| Residual | 3962 | 1465.08 | 0.86019 |  |  |
| Total | 3980 | 1703.2 | 1 |  |  |

1. Impact of the sequencing platform

*Equation: dist ~* Sequencing depth *+ sample age group + sequencing platform*

|  | Df | Sum Of Sqs | R2 | F | Pr(>F) |
| --- | --- | --- | --- | --- | --- |
| Sequencing depth | 1 | 8.87 | 0.00521 | 23.176 | 0.001 |
| sample age group | 4 | 144.91 | 0.08508 | 94.685 | 0.001 |
| sequencing platform | 2 | 21.23 | 0.01246 | 27.742 | 0.001 |
|  |  |  |  |  |  |
| Residual | 3973 | 1520.08 | 0.89249 |  |  |
| Total | 3980 | 1703.2 | 1 |  |  |

1. Impact of the bead beating extraction

*Equation: dist ~* Sequencing depth *+ sample age group + bead beating*

|  | Df | Sum Of Sqs | R2 | F | Pr(>F) |
| --- | --- | --- | --- | --- | --- |
| Sequencing depth | 1 | 4.74 | 0.00278 | 12.344 | 0.001 |
| sample age group | 4 | 146.6 | 0.08607 | 95.433 | 0.001 |
| bead beating | 1 | 15.16 | 0.0089 | 39.479 | 0.001 |
|  |  |  |  |  |  |
| Residual | 3974 | 1526.15 | 0.89605 |  |  |
| Total | 3980 | 1703.2 | 1 |  |  |

1. Impact of the sampling region

*Equation: dist ~* Sequencing depth *+ sample age group + region*

|  | Df | Sum Of Sqs | R2 | F | Pr(>F) |
| --- | --- | --- | --- | --- | --- |
| Sequencing depth | 1 | 3.54 | 0.00208 | 9.356 | 0.001 |
| sample age group | 4 | 135.16 | 0.07936 | 89.193 | 0.001 |
| region | 2 | 36.13 | 0.02121 | 47.681 | 0.001 |
|  |  |  |  |  |  |
| Residual | 3973 | 1505.18 | 0.88374 |  |  |
| Total | 3980 | 1703.2 | 1 |  |  |

1. Impact of the technical variables

*Equation: dist ~ Sequencing depth + sample age group + study name + region + bead beating + sequencing platform*

|  | Df | SumOfSqs | R2 | F | Pr(>F) |
| --- | --- | --- | --- | --- | --- |
| Sequencing depth | 1 | 0.68 | 0.0004 | 1.8397 | 0.01 |
| sample age group | 4 | 120.76 | 0.0709 | 81.6627 | 0.001 |
| study name | 9 | 29.54 | 0.01734 | 8.8779 | 0.001 |
| region | 0 | 0 | 0 | Inf |  |
| bead beating | 0 | 0 | 0 | Inf |  |
| sequencing platform | 1 | 0.72 | 0.00042 | 1.9409 | 0.012 |
|  |  |  |  |  |  |
| Residual | 3961 | 1464.36 | 0.85977 |  |  |
| Total | 3980 | 1703.2 | 1 |  |  |

**B: Including infant samples**

*Equation: dist ~ Sequencing depth + infant age + study name + born method + feeding practice + ABx exposures*

|  | Df | SumOfSqs | R2 | F | Pr(>F) |
| --- | --- | --- | --- | --- | --- |
| Sequencing depth | 1 | 0.59 | 0.0004 | 1.5948 | 0.029 |
| Infant age | 1 | 12.37 | 0.0084 | 33.2793 | 0.001 |
| Study name | 13 | 65.38 | 0.04441 | 13.5292 | 0.001 |
| Born method | 1 | 9.25 | 0.00628 | 24.8741 | 0.001 |
| Feeding practice | 6 | 25.15 | 0.01708 | 11.2746 | 0.001 |
| Abx exposures | 1 | 2.21 | 0.0015 | 5.9464 | 0.001 |
|  |  |  |  |  |  |
| Residual | 3473 | 1290.98 | 0.87701 |  |  |
| Total | 3496 | 1472.02 | 1 |  |  |

*Explanation for PERMANOVA analyses.

Separate PERMANOVA analyses for study_name (#1), sequencing platform (#2), geography (#3), and bead_beating (#4) were conducted because these variables show perfect collinearity. As demonstrated in our complete PERMANOVA model (#5), when all variables are included together, both region and bead_beating show complete nesting within study_name (R² = 0, F = Inf), indicating they are perfectly confounded with study effects. Therefore, analyzing these variables in separate models provides a clearer understanding of their contributions to community variation without the confounding effects of their complete nesting within studies.

To further investigate this, we performed a nested model comparison using ANOVA() between a model containing the methodological variables (nb_reads, sample_type, region, bead_beating) and a model that also included study_name. This analysis showed that study_name explained only an additional 1.6% of variance (difference in R² between 0.107 and 0.091) beyond what was explained by methodological variables. While this difference was statistically significant (p = 0.001), its small magnitude suggests that most of the study-related variation can be attributed to the methodological variables we recorded, supporting our approach of analyzing these variables separately to understand their contributions to community variation**.**

**Table S11 Top 10 bacterial species in the CARD database**

| Bacterial species | ARGs count |
| --- | --- |
| Pseudomonas_aeruginosa | 766 |
| Acinetobacter_baumannii | 589 |
| Klebsiella_pneumoniae | 511 |
| Escherichia_coli | 500 |
| Citrobacter_freundii | 115 |
| Enterobacter_cloacae | 108 |
| Campylobacter_jejuni | 76 |
| Acinetobacter_pittii | 62 |
| Proteus_mirabilis | 57 |
| uncultured_bacterium | 56 |
